# Recent Advances in Aggregation-Induced Emission Chemosensors for Anion Sensing

**DOI:** 10.3390/molecules24152711

**Published:** 2019-07-25

**Authors:** Ming Hui Chua, Kwok Wei Shah, Hui Zhou, Jianwei Xu

**Affiliations:** 1 Institute of Materials Research and Engineering, A*STAR (Agency for Science, Technology and Research), 2 Fusionopolis Way, Innovis, #08-03, Singapore 138634, Singapore; 2 Department of Building, School of Design and Environment, National University of Singapore, 4 Architecture Drive, Singapore 117566, Singapore; 3 Department of Chemistry, National University of Singapore, 3 Science Drive 3, Singapore 117543, Singapore

**Keywords:** aggregation–induced emission (AIE), anion, chemosensor, detection, sensing

## Abstract

The discovery of the aggregation-induced emission (AIE) phenomenon in the early 2000s not only has overcome persistent challenges caused by traditional aggregation-caused quenching (ACQ), but also has brought about new opportunities for the development of useful functional molecules. Through the years, AIE luminogens (AIEgens) have been widely studied for applications in the areas of biomedical and biological sensing, chemosensing, optoelectronics, and stimuli responsive materials. Particularly in the application of chemosensing, a myriad of novel AIE-based sensors has been developed to detect different neutral molecular, cationic and anionic species, with a rapid detection time, high sensitivity and high selectivity by monitoring fluorescence changes. This review thus summarises the recent development of AIE-based chemosensors for the detection of anionic species, including halides and halide-containing anions, cyanides, and sulphur-, phosphorus- and nitrogen-containing anions, as well as a few other anionic species, such as citrate, lactate and anionic surfactants.

## 1. Introduction 

Fluorescent chemosensors offer the main advantage of convenience: the detection of a particular species of interest can be performed on-site, rapidly, with obvious fluorescence changes that indicate the presence or absence of the analyte. Furthermore, these chemosensors often have low production costs, and are available in convenient-to-handle mediums such as one-off-use paper probes. The use of fluorescent chemosensors thus ideally avoids the hassle of performing traditional sensing methods through instrumental analysis, which are often costly and time-consuming. Therefore, over the past few decades, there has been in-depth interest and intensive research on the development of chromogenic and fluorescent chemosensors for environment and biological sensing, with a wide range of analytes spanning from neutral molecules, biomolecules to simple cationic and anionic ions [1,2,3,4,5,6,7,8,9,10,11,12,13,14]. Fluorescent chemosensors developed in the earlier days were, however, based on traditional luminogens, which exhibit aggregation-caused quenching (ACQ) properties [15]. ACQ luminogens, for example, pyrene, are structurally planar, which favourably aggregate via strong π-π interactions at the high concentration and unsolvated state. Together with collisional interactions among excited molecules and ground-state molecules, the formation of excimers and exciplexes due to π-π interaction leads to the effective quenching of fluorescence via non-radiative deactivation pathway [15]. ACQ luminogens are only emissive in dilute solution but non-emissive in concentrated solution or in the solid state. This imposes limitations to ACQ luminogens-based fluorescent chemosensory in practical applications, and even compromises their sensitivity in the event that they exhibit inherently weak emission in dilute solution. 

**Figure 1:**
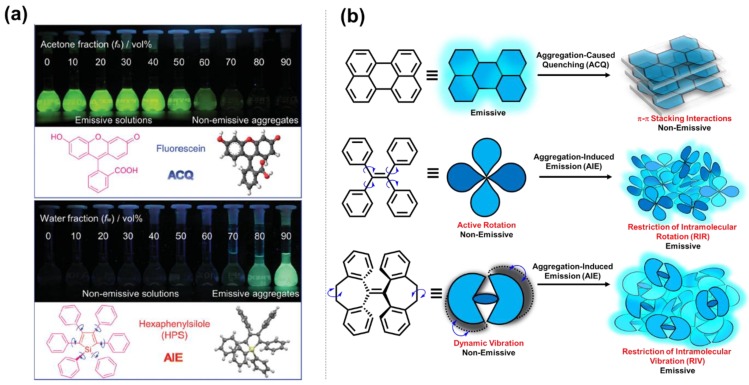
(**a**) Photographs showing the ACQ behaviour of fluorescein and AIE properties of HPS, in the mixture of water-miscible organic solvent and water. Reproduced with permission from [16]. Copyright 2014, John Wiley & Sons, Inc. (**b**) Schematics showing the general mechanism of ACQ and AIE.

In the early 2000s, a new class of luminogens, which included tetraphenylethylene (TPE), 9,10-distyrylanthracene (DSA) and heptaphenylsilole (HPS), were discovered to exhibit properties opposite to those of ACQ. These luminogens appear non-emissive in dilute solution but brightly emissive in the aggregated and unsolvated state. The term aggregation-induced emission (AIE) was thus coined for the observed phenomenon [16,17,18,19,20]. Similarly, the term aggregation-induced enhanced emission (AIEE) is used to describe the observation in which luminogens inherently exhibit weak emission in the solution state but greatly enhanced emission in the aggregation state. Figure 1a shows the difference in emission properties between an ACQ luminogen (fluorescein), and an AIEgen (HPS). Both luminogens are organo- soluble and insoluble in water. In solution, fluorescein exhibits green emission, whereas HPS is non-emissive. With the increase of proportions of water in a solution with a constant concentration of luminogen, both fluorescein and HPS form aggregates, but the emission of fluorescein is quenched, and a green emission of HPS is turned on. 

Detailed mechanistic studies into the luminescence properties of AIEgens strongly suggest that the AIE phenomenon arises from the restriction of intramolecular motion (RIM). A common similarity in the chemical structure of AIEgens such as TPE, DSA and HPS is that they all consist of multiple phenyl rings decorated over a small ethylene rod or an aromatic core. These rings do not remain static when AIEgens molecules are solvated, rotating about the C–C bond like rotor blades on a fan, consuming energy and thus leading to a non-radiative deactivation pathway for photo-excited AIEgens. AIEgens thus appear non-emissive in solution. Upon aggregation, however, the close-packing of AIEgens molecules leads to the restriction of intramolecular rotation (RIR), thus effectively halting the emission-quenching non-radiative deactivation pathway. In addition, the steric hindrance imposed by closely-attached rotatable groups forces AIEgens to adopt non-planar, twisted molecular structures, contributing to the aversion of detrimental π-π stacking upon aggregation. As such, non-radiative deactivation pathways are avoided and the AIEgens are emissive in the aggregated state [21]. 

More recently, several luminogens without rotatable aryl groups were also found to be AIE- or AIEE-active. These include 10,10′,11,11′-tetrahydro-5,5′-bidibenzo[*a,d*][7]annulenylidene (THBA), 5,5′-dibenzo[*a,d*][7]annulenylidene (BDBA) and Troger’s base containing Λ-shaped pyridinium salt 2,8-(6H,12H-5,11-methanodibenzo[*b,f*]diazocineylene)-di(p-ethenyl-N-methyl-pyridinium) ditosylate (DMDPS) [22,23]. Two highly-fused systems containing cyclooctatetraene (COT) cores to exhibit AIE and AIEE properties were also separately reported by Iodya et al. and Yamaguchi et al. [24,25]. Mechanistic studies show that intramolecular vibration and ring inversion in these luminogens play a dominant role in non-radiative deactivation of excited state. Aggregation, however, results in the restriction of intramolecular vibration (RIV) in these non-planar molecules and thus effectively turns on or enhances emission. Figure 1b shows the general mechanism of AIE phenomenon including RIM, RIR and RIV. 

AIE has attracted intensive attention from many research groups as evident in the exponential rise of publication counts over the years in this field. AIE luminogens have been reported for a wide range of applications, namely in bioimaging and biosensing [26,27,28,29,30,31], optoelectronics [32], chemosensing [33,34], and in stimuli-responsive materials [35,36]. In particular, AIEgens were extensively developed and employed for chemical sensing, from ions to harmful substances. AIEgens for chemosensing must be intelligently engineered and designed to cater for a specific mode of interaction with analytes, such as these bearing chelating, pH-sensitive and chemically reactive groups. The incorporation of AIE-active groups in chemosensing not only overcomes some limitations faced by ACQ-based fluorescence sensors as discussed earlier, but also affords the advantages such as high sensitivity, amplified emission-response in aqueous medium, and solid-state test strips. 

Anions play important biological roles in our body. Anions also exist at large in our environment, some of which are extremely harmful to our body. It will thus be vital to detect these anions in our surrounding environment, particularly harmful ones, to safeguard our health and safety. There has been extensive research over the decades in the development of anion fluorescent and colorimetric probes for easy and rapid sensing of anions [5,37,38,39]. In general, several characteristics of anions need to be considered in the development of fluorescent probes: (1) nucleophilicity, (2) basicity, and (3) sites available for coordination or supramolecular interactions. To begin with, some anions are highly nucleophilic and hence reactive towards electron-deficient functional groups. Fluorescent probes for selective detection of such anions often encompass reactive groups, which enable rendering of a change in optical and emission properties upon nucleophilic reaction with anions of interest. On the other hand, some anions preferentially act as a base and are possible to extract off acidic protons from fluorescent sensors, which may render changes in solubility or lead to further chemical reactions. Finally, some anions are able to act as mono- or multivalent ligands that coordinate with metal centres to readily form hydrogen bonds or engage in other forms of supramolecular interactions like anion-π and host-guest interactions, which can be taken into consideration in the development of fluorescent probes for selective detection of anions. 

While several reviews have been summarized the work on AIE-based chemical- and bio-sensors, in general, one that focuses solely on the detection of anions has yet to be undertaken. Therefore, in this review, we will timely review recent efforts starting from 2014 in the area of AIE-based organic fluorescent sensors for the detection of various anions including: (1) halides and halide-containing anions, (2) cyanides, (3) sulphur-containing anions, (4) phosphorus-containing anions, (5) nitrogen-containing oxoanions, and (6) others. Due to the different nature of each anionic species, different mechanisms may be involved in the detection of AIE-based sensors, which are summarised (but not limited to) as follows:

(1) sensor with a specific functional group reacts readily with anion of interest to cause changes in either colour or emission. This may be due to (i) change in extent of electronic conjugation, (ii) disruption to intramolecular electron and charge transfer processes, and/or (iii) change in solubility in an aqueous medium, leading to aggregation/disaggregation and hence emission turn-on/off. 

(2) Sensor deprotonated by anion of interest causes changes in emission due to (i) further chemical reactions, (ii) change in solubility in aqueous medium or (iii) intramolecular electron and charge transfer processes.

(3) Changes in colour and emission of sensors are induced by anion exchange at cationic centres.

(4) Preferential coordination, hydrogen bonding or supramolecular interactions between sensor and anion of interest causes changes in colour and emission. This may be due to (i) self-assembly due to coordination or supramolecular polymeric network; (ii) disassembly or disruption/changes to existing self-assembled structure hence an emission change/turn-off; or simply (iii) changes in electron and charge influences on the sensors.

## 2. Sensing of Halides and Halogen-Containing Anions

Halides and halogen-containing anions constitute an important class of anions due to their occurrence in the environment, useful industrial applications, and their vital roles in biological functions. The development of fluorescent sensors to detect these halides and halogen-containing anions has generated great interest amongst scientists. In this section, AIE-based fluorescent sensors serving such a function will be summarized. Amongst the halides, AIE-based fluoride chemosensors are well studied, whereas bromide and astatine sensors are rarely reported. In addition, AIE-based fluorescent sensors for the selective detection of chloride, hypochlorite, perchlorate and iodide are also summarized in this section.

### 2.1. Sensing of Fluorides

**Scheme 1:**
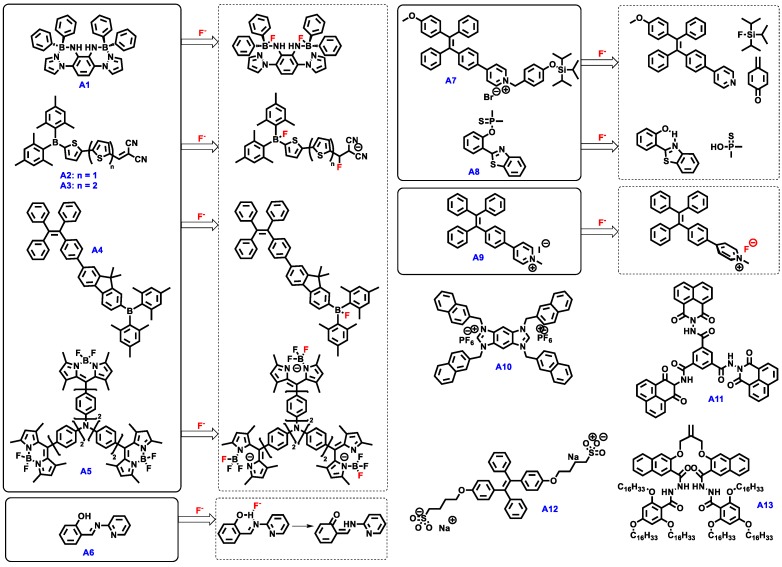
Chemical structures of AIE-based fluoride chemosensors (**A1**–**A13**) with selected sensing mechanisms shown.

Amongst the halides, fluoride is the smallest with the highest charge density. Fluoride is perhaps most well-known for its anti-cavity properties, which makes it commonly found in toothpaste and other oral-health products. In addition, fluoride is also found to be useful in the treatment of osteoporosis. Despite its health benefits, over-exposure to fluoride can lead to serious health issues, due to its ease of absorption and difficulty in excretion, thus detrimental to the kidneys, bones and teeth. In some countries, fluoride contamination in the groundwater source is a serious issue. Conventional means of fluoride sensing involves instrumental analysis such as ^19^F nuclear magnetic resonance, ion electrodes and other spectrophotometry [40,41,42]. On the other hand, fluorescence fluoride chemosensors have been shown to be more convenient and less expensive, and even more compatible with intracellular monitoring, leading to a large number of fluorescent and colourimetric fluoride chemosensors over the years [42]. In recent years, quite a significant number of AIE-based fluoride chemosensors were reported. In general, the common sensing mechanisms involve chemical reactions with fluoride, hydrogen bonding, and deprotonation, as well as supramolecular self-assembly. Scheme 1 summarises the chemical structures of these chemosensors (**A1**–**A13**) and their sensing mechanisms. The optical properties and sensing performance of these chemosensors are summarised in Table 1. 

**Figure 2:**
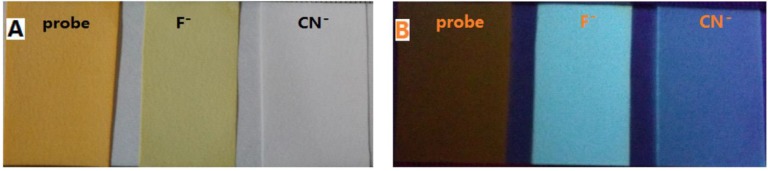
Colour change (**A**) and emission change (**B**) changes of **A5**-coated test strips upon exposure to fluoride and cyanide. Reproduced with permission from [46]. Copyright 2016, Elsevier B. V.

Conjugated molecules containing boron centres are particularly sensitive to the attack of fluoride anions due to the interaction of fluoride’s lone pair with the empty p_z_ orbital of boron. Thus, boron-containing luminogens can provide a good framework to develop fluoride chemosensors by recording the changes in luminescence caused by the reaction with fluoride. Compounds **A1**–**A5** are examples of AIE-based fluoride chemosensors based on the reaction of fluoride anions at the boron centres. AIE-active **A1** emitted green fluorescence upon aggregation but it was quenched in the presence of fluoride, which attacked the two boron centres and cleaved the weak B–N coordination bond between boron centres and pyrazole moieties [43]. In addition to the dimesityl boron centre, the dicyanovinyl group in **A2** and **A3** served as an additional site for reaction with fluoride. Both AIE-active compounds, which were reported to selectively detect fluoride and cyanide, were eventually fabricated into paper test strips for convenient fluorescence and colorimetric detection of the two harmful anions in water [44]. Similarly, AIEgen **A4**, which emitted intense blue fluorescence on aggregation or in the solid state, experienced partial quenching of fluorescence and blue-shift of absorption in the presence fluoride due to photoinduced electron transfer (PET) and disruption of the *p*-π conjugation between dimesitylboron group and the rest of the molecule [45]. Boron centres of boron dipyrromethene (BODIPY) luminogens were also found to be susceptible to fluoride attack. Wang et al. reported AIE-active triphenylamine-BODIPY conjugate **A5** whose intense green emission was quenched in the presence of fluoride. Compound **A5** was also found to selectively detect cyanide, also due to anion binding at the boron centre [46]. Test strips fabricated with **A5** turned from dark yellow to light blue and then blue under UV lamp upon exposure to fluoride and cyanide, respectively (Figure 2). 

Fluorine atoms and fluoride are known to readily form hydrogen bonding and, in some cases, lead to deprotonation of acidic protons. Schiff base compounds are known to be AIE-active and can undergo keto-enol tautomerization [55,56]. Chemosensor **A6** exhibited bright yellow emission in the aggregated and solid states but was almost non-emissive in solution. The addition of fluoride into solution of **A6** resulted in green emission turn-on due to hydrogen bonding between fluoride and phenol’s -OH group, and subsequently led to its deprotonation and possible keto-enol tautomerization [47].

**Figure 3:**
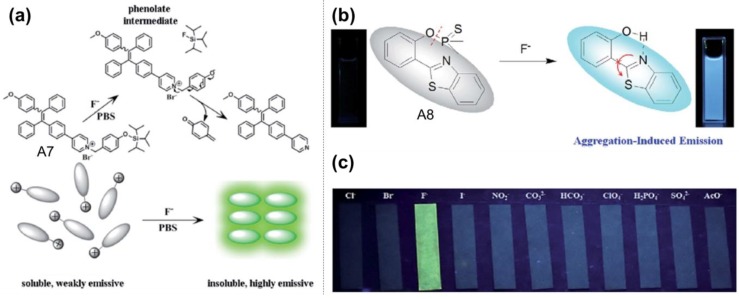
Mechanisms of fluoride sensing for **A7** (**a**) and **A8** (**b**). (**c**) Photo showing selective emission turn-on response of **A8**-coated paper test strips in the presence of fluoride. Reproduced with permission from [48,49]. Copyright 2016 and 2018, the Royal Society of Chemistry.

AIE-based chemosensors **A7** and **A8** underwent fluoride-promoted cleavage of specific groups in their respective molecules. **A7** structurally consisted of a TPE unit conjugated with a water-soluble pyridinium group. The presence of fluoride promoted the cleavage of triisopropylsily (TIPS) group on the pendant of pyridinium ring, resulting in a phenolate intermediate that underwent subsequent 1,6-elimination of *p*-quinone-methide, generating a water-insoluble TPE-pyridine aggregate (Figure 3a) and hence turning on green emission. [48] Similarly compound **A8** contained dimethylphosphinothionyl group which quenched fluorescence by inhibiting the excited state intramolecular proton transfer (ESIPT) process. Fluoride-promoted cleavage of the P–O bond generated highly blue-emissive 2-(2′-hydroxyphenyl)-benzothiazole due to the formation of intramolecular hydrogen bond (Figure 3b) [49]. High selectivity towards fluoride anions can be achieved for both chemosensors due to the selective bond cleavage reactions that take place only in the presence of fluoride anions. Paper test strips that were fabricated from **A8** are shown in Figure 3c, displaying the obvious fluorescence change. 

TPE-pyridinium compound **A9** is an interesting example of a fluoride chemosensor that is based on an anion exchange and subsequent structural conformational changes. The displacement of iodide in **A9** with fluoride at the pyridinium group in aqueous solution resulted in both blue-shifting of emission wavelength from 560 to 460 nm, as well as enhancement of blue emission upon decrease in π-conjugation between pyridinium and TPE moieties [50].

**Figure 4:**
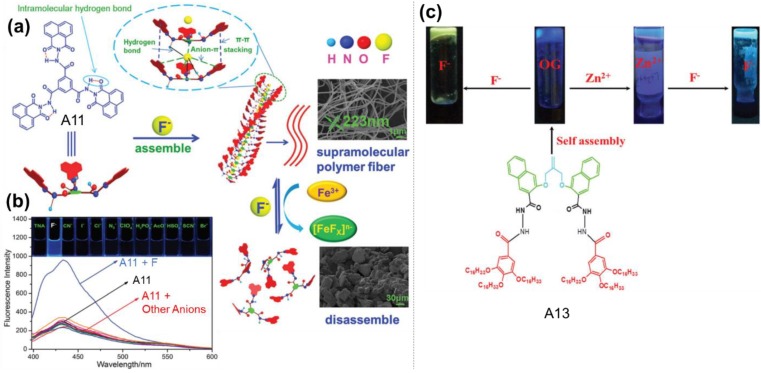
(**a**) Schematic representing the assembly and disassembly of **A11**-fluoride supramolecular polymer. (**b**) Emission spectra and photo showing the response of **A11** in the presence of fluoride and other anions. (**c**) Photos showing the emission colour of supramolecular gels formed between **A13** and fluoride and Zn^2+^. Reproduced with permission from [52,54]. Copyright 2018 and 2016, the Royal Society of Chemistry.

Making use of specific electrostatic, ionic and supramolecular interactions, fluoride-mediated aggregation/disaggregation, and supramolecular assembly/disassembly is an effective means for the design of fluoride chemosensors. Electrostatic interactions between fluoride and benzobisimidazolium derivative **A10** led to the formation of stable aggregates in acetonitrile and hence an AIE amplification of fluorescence [51]. Tripodal naphthalene imide sensor **A11** assembled into a network of supramolecular polymer fibre in the presence of fluoride due to anion-π interactions between the centre phenyl ring of **A11** and fluoride, and π-π stacking between the naphthalene imide side groups (Figure 4a) [52]. A resulting blue emission turn-on (Figure 4b) could be reversibly turned off via the addition of Fe^3+^ that promoted disassembly of the network. On the contrary, water-soluble TPE derivative **A12** readily formed blue-emissive insoluble aggregates in the presence of Al^3+^ based on ionic interactions. The addition of fluoride, however, induced disaggregation and hence an emission turn-off due to the preferential formation of AlF_3_ [53]. Organic supramolecular gelator **A13** exhibited different fluorescence responses upon gelating in the presence of Zn^2+^ and fluoride. As shown in Figure 4c, **A13** formed an AIE-active metallogel with Zn^2+^ exhibiting strong blue fluorescence. Introduction of fluoride thereafter changed the emission colour to blue-green without undergoing the gel-sol phase. However, addition of **A13** formed an AIE-active organogel with greenish-yellow fluorescence with fluoride in the absence of Zn^2+^ [54].

### 2.2. Sensing of Chlorides

**Scheme 2:**
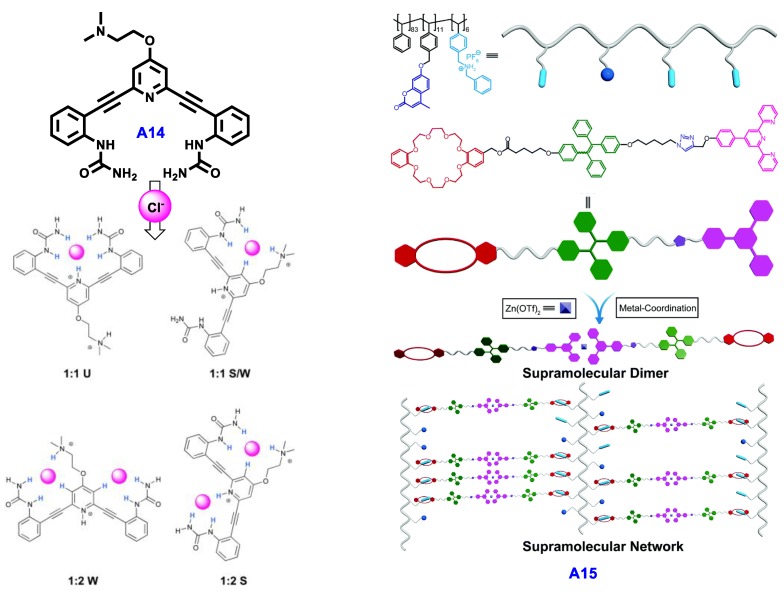
Chemical structures of AIE-based chloride chemosensors (**A14**–**A15**). Reproduced with permission from [58,59]. Copyright 2015 and 2017, the Royal Society of Chemistry.

Chloride serves as an important electrolyte in the human body, transporting into and out of cells through chloride channels, participating in maintaining cell homeostasis and nerve transduction. [57] In the environment, chloride exists as salts and minerals, some of which may be used for consumption as food seasoning and preservative. As compared to fluoride sensing, chloride chemosensors appear much rare, probably due to challenges in engineering selectivity towards anions, as well as the absence of adverse harmful effects that warrant an urgency to develop selective chloride chemosensors. The chemical structures and sensing mechanisms of two AIE-based chloride chemosensors are shown in Scheme 2 and their optical properties and sensing performances are summarised in Table 2. 

Pyridine biurea ligand **A14**, when protonated at pyridine’s nitrogen under acidic conditions, was found to selectively coordinate chloride in water. Coordination of chloride resulted in an AIE-active complex that aggregated in water, turning on bright green emission [58]. The intensity of turn-on emission caused by chloride was significantly greater than that caused by perchlorate and fluoride. Four modes of coordination between protonated **A14** and chloride were proposed as shown in Scheme 2. 

Supramolecular network **A15** was made up of Zn^2+^, a polystyrene-based polymer and a TPE-based molecule via metal coordination and host-guest interactions [59]. RIR of TPE in network **A15** causes it to emit bright blue fluorescence in the gel state. The presence of chloride, however, resulted in the disruption of the supramolecular network, hence, effectively turning off the blue emission [59]. The same was observed when triethylamine and cyclen were added separately as the supramolecular network was disrupted by these species added.

### 2.3. Sensing of Hypochlorite and Perchlorate

Many halogens are known to form halogen oxides and halogen oxoanions where halogen atoms take on different oxidation states. For instance, chlorine may be further oxidised into hypochlorite (ClO^−^), chlorate (ClO_3_^−^) and perchlorate (ClO_4_^−^). Amongst these, hypochlorite anion is an active ingredient in household bleach (sodium hypochlorite), and disinfectant in swimming pools (calcium hypochlorite). Hypochlorite anion is also one of the most important reactive oxygen species (ROS) generated by the body in mediating important pathological and biological processes, such as elimination of microbial for the former, and signal transduction and homeostasis for the latter. However, due to its strong oxidising power, over-exposure to hypochlorite and over-generation of it in the body may be harmful. The development of hypochlorite chemosensors would therefore be useful for studying biological functions and environment sensing. 

**Scheme 3:**
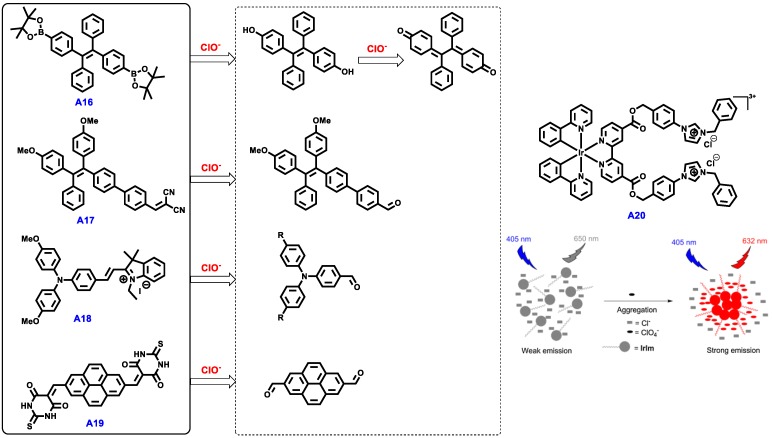
Chemical structures of AIE-based hypochlorite chemosensors (**A16**–**A19**) and perchlorate chemosensor **A20**, with their respective sensing mechanisms.

Due to the strong oxidizing power of hypochlorite, AIE-based hypochlorite chemosensors generally employ oxidative-type chemical reactions between the anion and selected functional groups on the chemosensor molecules, to cause a change in chemical structure and thus render an observable emission change. Scheme 3 shows the chemical structures of AIE-based hypochlorite sensors **A16**–**A19**, before and after reaction with hypochlorite. Their corresponding optical properties and sensing performance are summarised in Table 3. 

**Figure 5:**
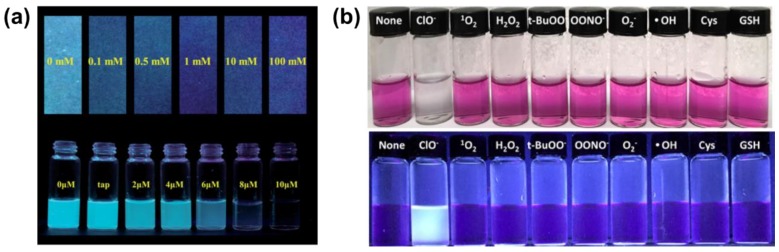
(**a**) Photos showing the emission-quenching of **A16** in aqueous solutions and **A16**-coated filter papers on exposure to water samples with an incremental amount of hypochlorite anions. Reproduced with permission from [60]. Copyright 2017, the Royal Society of Chemistry. (**b**) Photos showing the selective decolourisation and fluorescence turn-on of **A18** in aqueous solution on exposure to hypochlorite. Reproduced with permission from [62]. Copyright 2019, Elsevier B. V.

AIE-active compound **A16**, structurally comprising TPE functionalized with two boronic acid pinacol ester groups, is highly insoluble in water, and its nanoaggregates exhibit bright blue fluorescence. The presence of up to two molar equivalences of hypochlorite, however, converted the boronic esters into hydroxy groups, and addition of more hypochlorite, further oxidised the resultant dihydroxy-TPE into a more aqueous-soluble quinoidal form (Scheme 3), resulting in the deformation of nanoaggregates and hence a decrease in fluorescence intensity [60]. As shown in Figure 5a, both aqueous solutions and test strips made from **A16**-coated filter papers show obvious quenching of blue fluorescence upon exposure to hypochlorite anions.

Highly oxidizing hypochlorite anions could also mediate oxidative cleavage reactions to certain functional groups, and this strategy was employed in chemosensors **A17**–**A19** [61,62,63]. Hypochlorite-mediated oxidative cleavage eventually afforded products with formyl groups for all three compounds, which naturally lead to observable emission changes. Aggregates of **A17** and surfactants in aqueous medium changed from bright red to weak green emission, and this could be used for the ratiometric imaging of endogenous hypochlorite in zebrafish and live cells [61]. The indole group in **A18** was cleaved after the reaction and there was no twisted intramolecular charge transfer (TICT) in its reaction product, and an blue-green emission turn-on was observed (Figure 5b) [62]. Similarly, due to hypochlorite-mediated oxidative cleavage, strong TICT (or ICT) between pyrene and 2-thioxo-dihydropyrimidine-4,6-dione groups in the reaction product of **A19** no longer existed, which eventually caused the enhancement of blue emission of **A19** aggregates [63]. 

Perchlorate contamination in food, water sources, and the environment is a serious problem because the harmful anion can lead to thyroid inhibition by interfering with iodide uptake from the bloodstream into the thyroid gland. The timely and sensitive detection of perchlorate is thus of great importance to safeguard human health. Chao et al. reported an iridium(III) complex, **A20**, as an AIE-based perchlorate sensor, whose structure is shown in Scheme 3 [64]. Water-soluble **A20** exhibited no emission in aqueous medium but formed aggregates in the presence of perchlorate, resulting in a turn-on of red emission. 

### 2.4. Iodide Sensing 

**Scheme 4:**
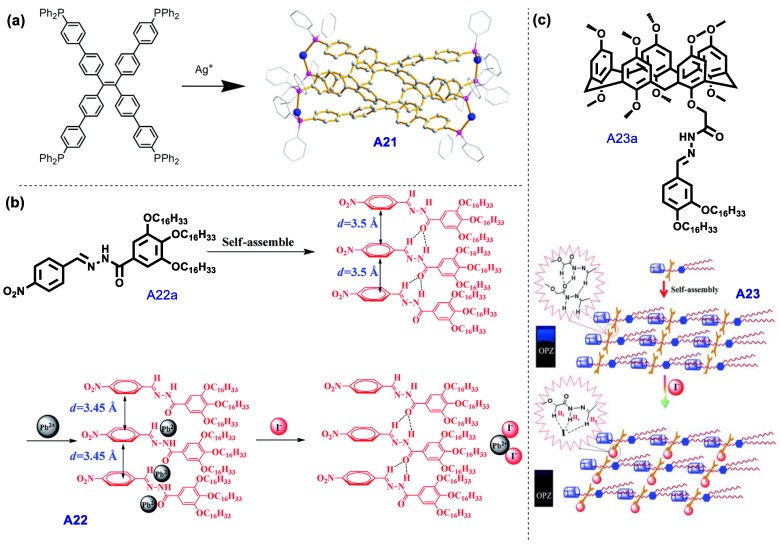
Chemical structures of AIE-based iodide chemosensors **A21** (**a**)**, A22** (**b**) and **A23** (**c**), with selected sensing mechanisms (for **A22** and **A23**) shown in (**b**) and (**c**), respectively. Reproduced with permission from [66,67,68]. Copyright 2016 and 2017, the Royal Society of Chemistry.

Iodine is necessary for normal biological functions in trace amount. For instance, they are required by the thyroid gland to produce thyroid hormones. Iodine deficiency can lead to intellectual disability, and affect normal thyroid functions. However, an excessive intake of iodine can also lead to goiter, hypo- and hyperthyroidism. Scheme 4 shows the structure and mechanism of AIE-based iodide sensors **A21**–**A23**, whereas their optical properties and sensing performance summarised in Table 4. 

Zhang et al. reported a silver–phosphine tetragonal cage, **A21**, formed by the coordination of Ag^+^ with a TPE-based tetradentate phosphine ligand, which emitted bright green luminescence both in solution and solid state due to RIR mechanism of the TPE moiety [66]. Ionic interaction between iodide and Ag^+^ centres, however, resulted in the quenching of this emission, making **A21** a promising candidate for iodide chemosensing. Two iodide-responsive supramolecular organogel systems, **A22** and **A23**, were separately reported by Wei et al. The organogelator **A22a** (Scheme 4b) could self-assemble with Pb^2+^ to form organogel **A22**, which exhibited blue fluorescence. Treatment of **A22** with iodide ions can reversibly switch off the fluorescence under gel–gel state by competitively binding with Pb^2+^ leading to changes in interlamellar supramolecular interactions between **A22** and a reduction of π-π stacking distance [67]. Similarly, pillar [5]arene-based organogelator **A23a** can self-assemble into stable organogel **A23** via a series of supramolecular interactions, including hydrogen bonding and host–guest interactions (Scheme 4c), which exhibited blue fluorescence [68]. Treatment with iodide in the gel–gel state disrupted hydrogen bonding and hence assembly of gel **A23**, leading to an 80% decrease in fluorescence intensity. The detection limits of **A22** and **A23** were determined to be 0.1 μM and 94 nM, respectively, and their iodide-responsive capabilities make them useful as potential fluorescent security display materials [67,68].

## 3. Sensing of Cyanide

**Scheme 5:**
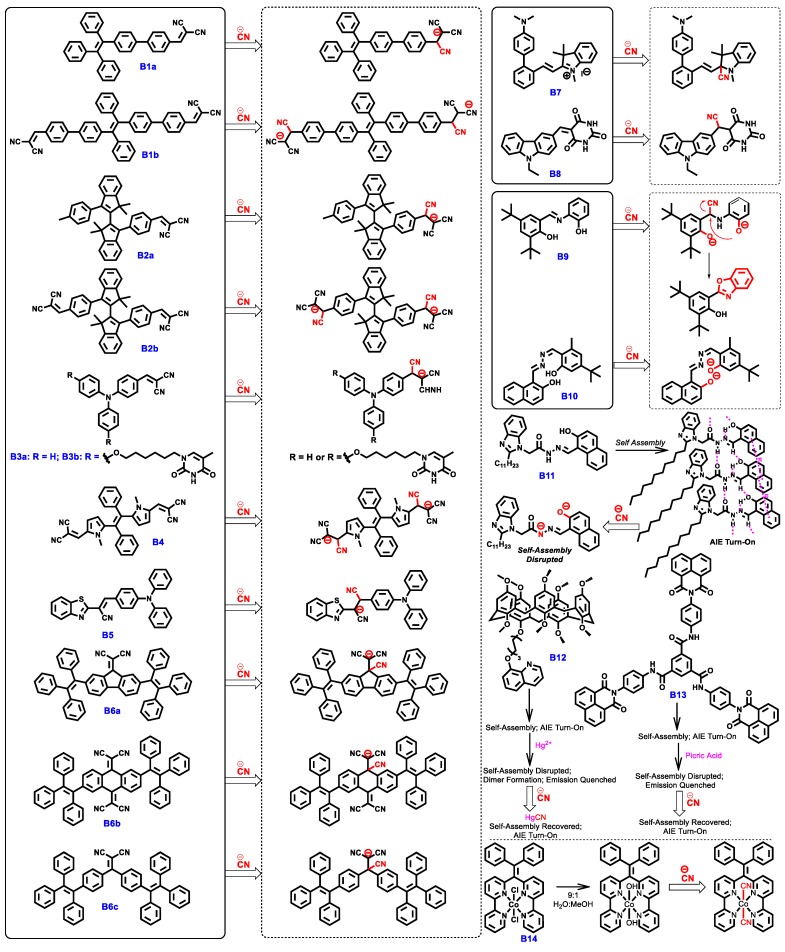
Chemical structures and AIE-based cyanide chemosensors (**B1a**–**B14**) and their sensing mechanisms.

Pseudohalides are anions in that their chemistry and reactivity resemble that of halides. Amongst the pseudohalides, sensing of cyanides receives the most attention due to its high toxicity. The lethal dose of cyanide to human beings is 1.5 mg/kg of body weight and unfortunately, cyanide contamination in the environment remains a real threat, particularly in the mining industries. To date, a large number of novel colorimetric and fluorescence cyanide chemosensors have been developed [69,70,71,72]. The common mechanisms for cyanide sensing involve either coordination of cyanide anions, or chemical reaction with cyanide nucleophile. These strategies are also evident in numerous examples of AIE-based cyanide chemosensors discussed in this section. The chemical structures and general mechanism of the fluorescent chemosensors **B1a**–**B14** are given in Scheme 5, and their optical properties and sensing performances are summarized in Table 5.

The general strategy in designing cyanide chemosensors is the incorporation of dicyanovinyl groups into chemical structures of chemosensors, which can be achieved by performing a simple Knoevenagel condensation between aldehyde intermediates and malanonitrile. Each of the AIE-based cyanide chemosensors **B1a**–**B4** contains one to two dicyanovinyl groups functionalized with an AIE-active moiety, e.g., TPE or triphenylamine. Cyanide nucleophiles would readily attack the electron deficient vinyl carbon centre of the dicyanovinyl group adjacent to the two cyano groups, forming an anion with negative charge stabilised on the adjacent vinyl carbon, which may or may not undergo subsequent protonation. The general consequences for a nucleophilic addition of cyanide to the dicyanovinyl group would be: (1) the disruption of electronic conjugation and ICT between the rest of the molecule with the two electron withdrawing cyano groups, hence a change in colour and emission properties; and (2) an increase in aqueous-solubility of the anionic cyanated product, and hence an emission change.

**Figure 6:**
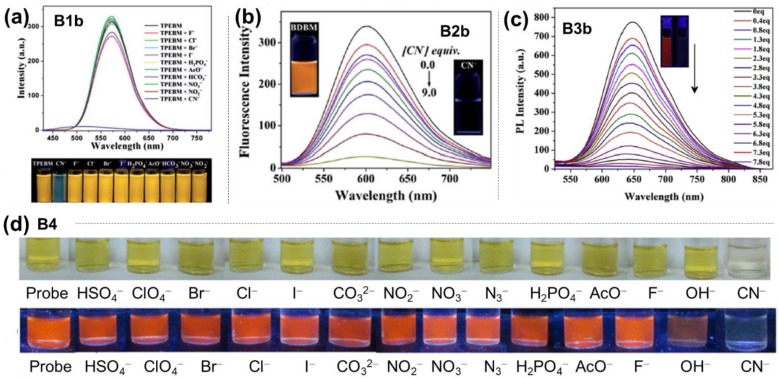
(**a**) Photoluminescence spectra and photos showing the selective quenching of orange fluorescence of **B1b** in aqueous solution. Reproduced with permission from [73]. Copyright 2014, the Royal Society of Chemistry. (**b**) Spectrofluorometric titration of **B2b** with cyanide with photos showing quenching of orange emission. Reproduced with permission from [74]. Copyright 2015, the Royal Society of Chemistry. (**c**) Spectrofluorometric titration of **B3b** with cyanide with photos showing quenching of red emission. Reproduced with permission from [76]. Copyright 2016, Elsevier B. V. (**d**) Photos showing the selective decolourisation and quenching of orange emission of **B4** by cyanide. Reproduced with permission from [77]. Copyright 2017, Wiley-VCH Verlag GmbH & Co. KGaA.

AIE-active chemosensors **B1a** and **B1b** formed aggregates in aqueous solution containing CTAB surfactant, exhibiting greenish-yellow and orange emission, respectively. An emission turn-off was observed in the presence of cyanide due to nucleophilic addition of cyanide at the dicyanovinyl groups which resulted in disaggregation (Figure 6a) [73]. Similarly, the yellowish orange and orange fluorescence arising from the aggregation of chemosensors **B2a** and **B2b** was quenched in the presence of cyanide due to the same principle described above (Figure 6b) [74]. AIE-active dicyanovinyl-triphenylamine based chemosensor **B3a** was reported to detect both cyanide and sulphite anions with a detection limit of 9.88 nM and 0.107 μM, respectively, in aqueous media [75]. On the other hand, addition of two pendant arms carrying thymine groups to dicyanovinyl-triphenylamine allowed derivative **B3b** to detect Hg^2+^ via an observable turn-on of red emission in addition to cyanide detection, which exhibited the opposite quenching of red-emission (Figure 6c) [76]. Also reported was the full quenching of orange emission of **B4** aggregates in aqueous solution in the presence of cyanide due to the formation of aqueous soluble cyanated species, leading to disaggregation of the AIE-active compound (Figure 6d) [77]. In general, this sensing mechanism combining nucleophilic attack of cyanide and a change in aqueous-solubility of the cyanated species allows for sensitive detection of cyanide in aqueous medium, and hence is applicable to cyanide sensing of water sources where contamination may be an issue in some parts of the world.

Functional groups that structurally resemble dicyanovinyl can also be useful in the development of novel cyanide chemosensors. Cyanide nucleophiles were able to attack the electron-deficient carbon on the cyanoethylene group of **B5**, effectively breaking electronic conjugation between triphenylamine and benzothiazole moieties, and rendering the anionic cyanated species water-soluble. This led to disaggregation and turn-off of orange emission in full aqueous medium [78]. The reported detection limit of 0.22 μM was lower than the maximum permissible level of cyanide in water of 1.9 μM as set by the World Health Organization (WHO). 1,1-Dicyanomethylidene groups in compounds **B6a**, **B6b** and **B6c** were also equally sensitive to cyanide nucleophile attack. Interestingly, upon aggregation, **B6a**, **B6b** and **B6c** were initially totally non-emissive, weakly emissive in solid state, and highly emissive, respectively. The cyanated species of **B6a** and **B6c** were found to be protonated and were AIE-active whereas cyanated **B6b** appeared chemically unstable. Paper probes coated with **B6a** revealed an emission turn-on whereas highly emissive **B6c**-coated paper probes revealed emission turn-off upon application of cyanide solutions [79].

**Figure 7:**
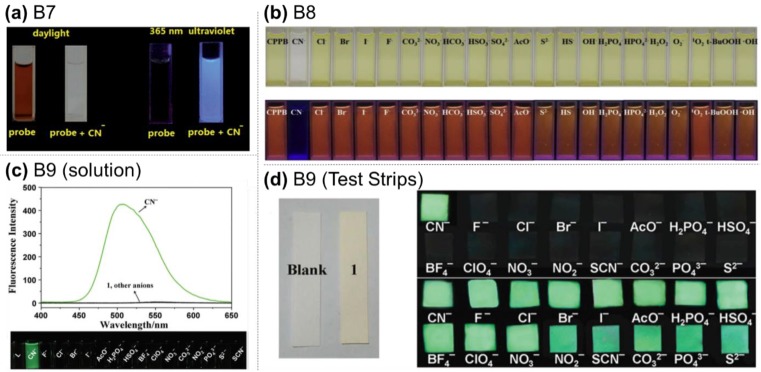
(**a**) Photos showing colour and fluorescence change of **B7** in water before and after addition of cyanide. Reproduced with permission from [80]. Copyright 2016, the Royal Society of Chemistry. (**b**) Photos showing the selective decolourisation and emission-quenching of **B8** in aqueous solution by cyanide. Reproduced with permission from [81]. Copyright 2019, Elsevier B. V. (**c**) Photoluminescence spectra and photos of **B9** in the presence of different anions. (**d**) Photos showing the colour of **B9**-coated test papers, and its emission test papers after exposure to various anions in the absence (above) and presence (below) of cyanide. Reproduced with permission from [82]. Copyright 2017, the Royal Society of Chemistry.

Nucleophilic attack of cyanide can also occur on other electron-poor functional groups, making these useful in the design of cyanide chemosensors, such as the indolium moiety of **B7** and ethylene-barbiturate group of **B8 [80,81]**. Nucleophilic attack of cyanide occurred at 2-position carbon of the indolium moiety in **B7**, whereas the same attack happened at the vinyl carbon of the C=C bond bridging barbiturate group and carbazole moiety in **B8**. It is worth noting that the cyanide-sensitive indolium group has been utilized in an earlier report of AIE-based cyanide sensor whereby it was attached to TPE via an ethylene bridge at the 2- position, and a green emission turn-on was observed upon positive detection of cyanide with a detection limit of 91 nM (vs. LOD of 55 nM for **B7**) [88]. A blue emission turn-on was observed for aqueous solution of **B7** upon exposure to cyanide due to the aggregation of the resultant insoluble cyanated adduct [80], whereas aggregate solution of **B8** revealed a turn-off of orange emission due to the disaggregation of soluble cyanated adduct (Figure 7a,b) [81].

The presence of hydroxy groups in AIE Schiff base chemosensors **B9**–**B11** revealed observable emission changes upon deprotonation by basic cyanide anions. For **B9**, the deprotonation of two OH groups and nucleophilic addition of cyanide at the C=N bond interestingly led to a subsequent cyclization reaction that formed a benzoxazole ring [82]. This resultant species was AIE-active, which resulted in a turn-on of green fluorescence both in aqueous medium and on test paper strips (Figure 7c,d). The deprotonation of two OH groups in **B10** by cyanide anions resulted in an emission colour change from yellow to orange, which was proposed to be attributed to ICT effect imposed by the -O^−^ group and possibly subsequent cyclization owing to charge separation [83]. Supramolecular gelator **B11** underwent self-assembly via an array of hydrogen bonding and π-π stacking, giving rise to an organogel with green fluorescence. The introduction of cyanide deprotonated the amide and hydroxy groups, leading to the inhibition of hydrogen bonding and hence disrupting self-assembly, and finally the emission colour of the organogel changed from green to blue as a result [84].

**Scheme 6:**
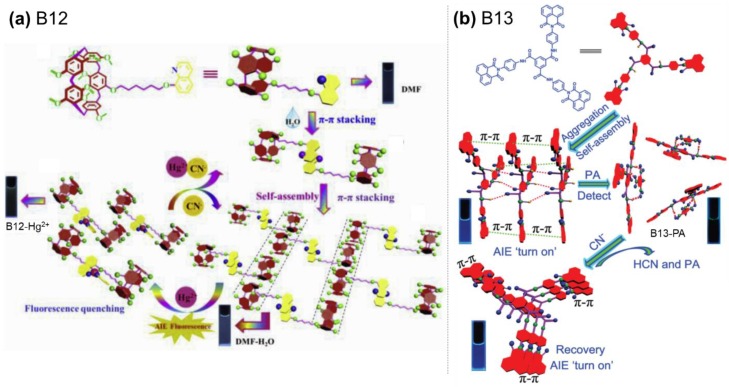
Illustrations of supramolecular self-assemblies of **B12** (**a**) and **B13** (**b**), the subsequent disruption to supramolecular network in the presence of Hg^2+^ and picric acid, respectively, and the eventual recovery of self-assembled network and blue fluorescence, on the addition of cyanide. Inset are photos showing the fluorescence colour of the composition at each stage. Reproduced with permission from [85,86]. Copyright 2019, Elsevier B. V. and the Royal Society of Chemistry.

Emission changes arising from the recovery of supramolecular self-assembled network as a result of addition of cyanide is another interesting mechanism for the sensing of cyanide. A more detailed schematic of their sensing mechanisms is shown in Scheme 6. The self-assembly of pillar[5]arene derivatives **B12** and tripodal compound **B13** via π-π stacking interactions turned on blue emission for both networks [85,86]. The addition of Hg^2+^ to supramolecular network of **B12**, and picric acid to that of **B13** disrupted the self-assembly resulting in the quenching of blue fluorescence. Interestingly, the blue fluorescence can be recovered via the introduction of cyanide, which selectively interacted with Hg^2+^ (to form Hg(CN)_2_) and picric acid (deprotonating it to form HCN) thereby reinstating the self-assembly of **B12** and **B13** [85,86]. **B12** and **B13** sensed Hg^2+^ and picric acid with a detection limit of 25.3 nM and 11.9 nM, respectively, and the subsequent compositions sensed cyanide with a detection limit of 77.1 nM and 0.745 μM, respectively [85,86].

Finally, by the coordination of cyanide to metal centres, an AIE-active cobalt (II) complex, **B14**, was reported to undergo ligand exchange in the presence of cyanide to its presence in aqueous medium. This resulted in a decrease of solubility of the cobalt complex and increased aggregation of, thereby turning on of blue emission. A low detection limit of 0.59 μM was reported from the sensing system [87].

## 4. Sensing of Sulphur-Containing Oxoanions

**Scheme 7:**
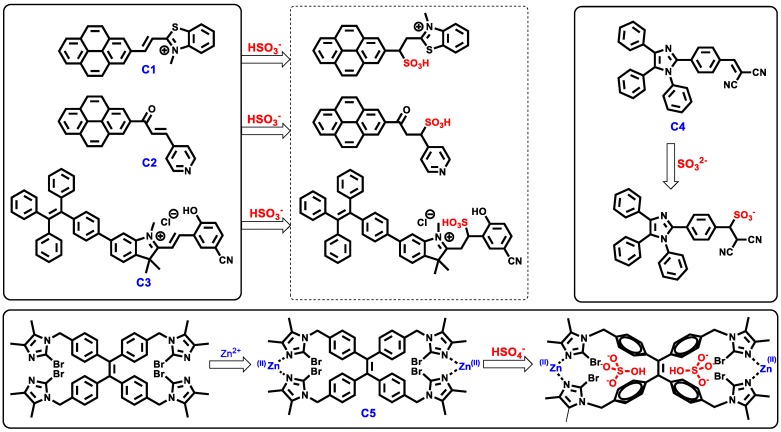
Chemical structures of AIE-based bisulphite, sulphite and bisulphate chemosensors **C1**–**C5**, and their general sensing mechanisms.

Sulphur forms oxoacids, for instance, sulphite (SO_3_^2−^), bisulphite (HSO_3_^−^), sulphate (SO_4_^2−^) and bisulphate (HSO_4_^−^) anions, which exist in aqueous solution or solid salts in which sulphur adopts different oxidation states. Some of these sulphur-containing oxoanions, such as HSO_3_^−^ and SO_3_^2^^−^, are toxic to human health. Sensing of sulphur-containing anions would be interesting although such chemosensors appear more rare compared to those for the detection of other anions. In this section, several AIE-based chemosensors for the sensing of bisulphite, sulphite and bisulphate will be summarized. The chemical structures and general mechanisms of these chemosensors are illustrated in Scheme 7, and their optical properties and sensing performances are summarised in Table 6.

### 4.1. Sensing of Bisulphite

Due to antioxidant and antimicrobial properties, bisulphite anions (HSO_3_^−^) can be used as a preservative for food and wine in the form of sodium bisulphite salt. Bisulphite salts are also commonly added to drugs like ephedrine to prevent undesirable oxidation reactions. Sodium bisulphite is also commonly used in wastewater treatment to reduce chlorine disinfectant prior to discharging into water bodies like reservoirs. Despite these applications, bisulphites are known to be toxic, and can lead to allergic reactions and diseases at a moderate to high level. In addition, the detection of bisulphite can also be an avenue for monitoring harmful sulphur dioxide in vivo and in vitro as it is known that sulphur dioxide exists in water as an equilibrium between sulphite and bisulphite anions at neutral pH.

**Figure 8:**
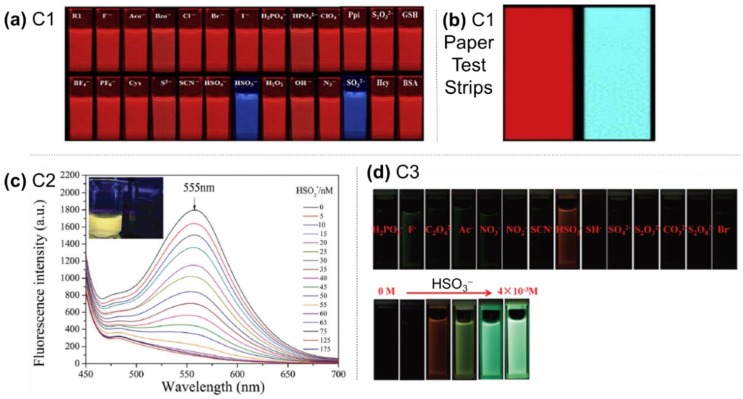
(**a**) Photos showing the selective emission colour change of **C1** solution due to HSO_3_^−^ and SO_3_^2^^−^. (**b**) Photos showing emission change of C1-coated paper test strips on exposure to HSO_3_^−^. Reproduced with permission from [89]. Copyright 2016, Elsevier B. V. (**c**) Spectrofluorometric titration of **C2** with HSO_3_^−^ with photos showing the quenching of yellow emission upon exposure to the anion. Reproduced with permission from [90]. Copyright 2018, the Royal Society of Chemistry. (**d**) Photos showing the selective turn-on of red emission of **C3** in the presence of low amount of HSO_3_^−^ and the emission change of **C3** solution on increasing amount of HSO_3_^−^ added. Reproduced with permission from [91]. Copyright 2019, the Royal Society of Chemistry.

Chemosensors containing Michael acceptors are good candidates for the detection of bisulphite anions [94,95,96,97,98,99,100]. Similarly, AIE-based bisulphite sensors **C1**–**C3** (Scheme 7) with Michael acceptors can readily undergo a Michael addition with bisulphite nucleophile (Scheme 7). **C1** in water/DMF (7:3 *v*/*v*) emitted blue in the presence of HSO_3_^−^ and SO_3_^2^^−^ anions (Figure 8a) due to the Michael addition of HSO_3_^−^ and SO_3_^2^^−^ at the C=C bond bridging pyrene and benzthiazlium moieties, effectively inhibiting ICT between them [89]. Paper test strips coated with compound **C1** also exhibited similar emission change (Figure 8b). Likewise, Michael addition of HSO_3_^−^ to the α,β-unsaturated ketone group of **C2** resulted in the breaking of conjugation between pyrene and pyridine moiety. The quenching of yellow emission of AIE-active **C2** in PBS buffer solution was observed (Figure 8c) [90]. TPE-derivative **C3** with a vinyl indolium group served as a Michael acceptor to detect HSO_3_^−^ in acidic aqueous condition [91]. The Michael addition reaction between **C3** and HSO_3_^−^ rendered hydrophilic insoluble product, leading to aggregation and hence turning on the emission from red to green with the increase of the concentration of HSO_3_^−^ (Figure 8d).

### 4.2. Sensing of Sulphite

Like bisulphite, sulphites (SO_3_^2^^−^) are commonly used in food preservation and enhancement, protecting food from oxidation and microbial contamination, but over-exposure to and consumption of sulphite may lead to adverse allergy reactions. SO_3_^2^^−^ is the deprotonated form of HSO_3_^−^ and is nucleophilic. Thus, chemosensors for the sensing of SO_3_^2^^−^ often contain electron-deficient functional groups to trigger a nucleophilic attack from the anion to activate a colour and emission response.

Both AIEgens **C4** (Scheme 7) and **B3a** (Scheme 5) contain dicyanovinyl groups that readily undergo nucleophilic addition with SO_3_^2^^−^ anions. Upon addition of SO_3_^2^^−^, the intense yellow emission of **C4** aggregate solution in the presence of CTAB surfactant was quenched, revealing a weak blue fluorescence due to the disruption of electronic conjugation between tetraphenylimidazole moiety and the two cyano groups, and the reduction in self-assembled nanoparticle size of the resulting species [92]. An ultrafast detection time of 15 s and ultralow detection limit of 7.4 nM were reported for **C4**. Similarly, the fluorescence of **B3a** aggregate was quenched in the presence of SO_3_^2^^−^ with a detection limit of 0.107 μM and a response time of 30 min [75].

### 4.3. Sensing of Bisulphate

Bisulphate (HSO_4_^−^) salts are commonly used in additives, cleaning agents and acidifier for treatment of swimming pools. Molina et al. reported a TPE-tetraimidazole ligand that selectively coordinates with Zn^2+^ cation to form ion-pair complex **C5**, where an increase in blue fluorescence by up to 18-fold was observed [93]. **C5** was found to selectively sense HSO_4_^−^ by acting as an ion-pair receptor whereby the anion was coordinated within the conformational-fitting cavities of **C5** (at the imidazole binding sites) in a 2:1 anion/receptor ratio with calculated binding constants of K_1_ = 2.2 × 10^5^ M^−1^ and K_2_ = 3.4 × 10^4^ M^−1^. An enhancement and blue-shifting of fluorescence was observed [93].

## 5. Sensing of Phosphorus-Containing Anions

**Scheme 8:**
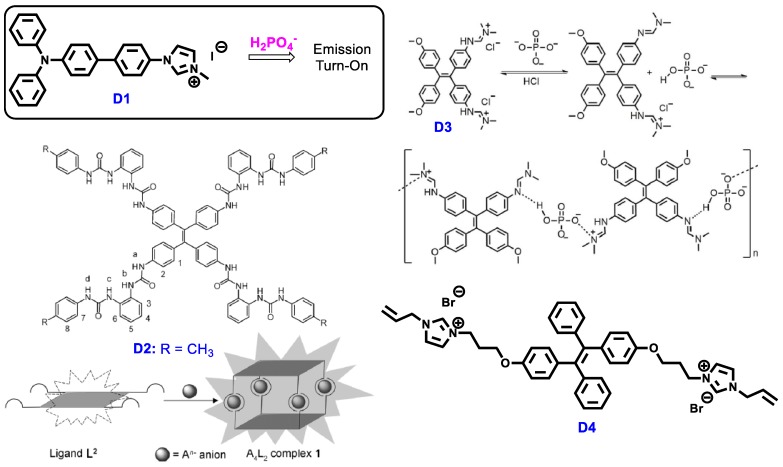
Chemical structures of AIE-based sensors for the phosphate sensing (**D1**–**D4**) and their sensing mechanisms. Chemical schemes for **D2** and **D3** reproduced with permission from [101,102]. Copyright 2014 and 2019, Wiley-VCH Verlag GmbH & Co. KGaA.

**Scheme 9:**
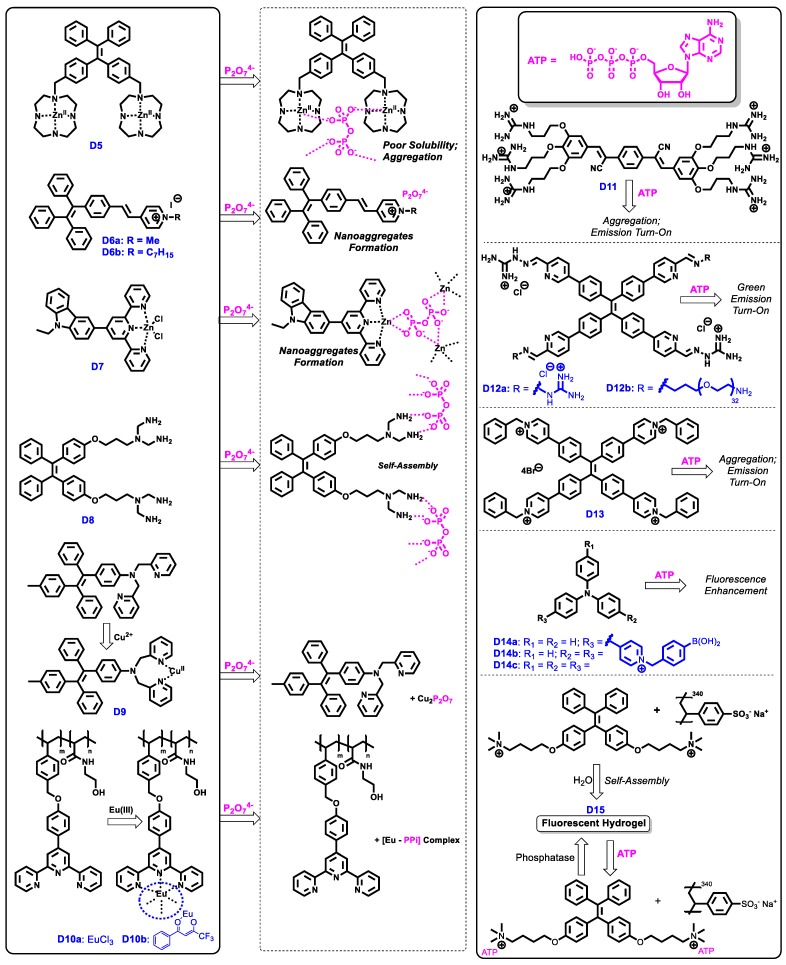
Chemical structures of AIE-based sensors for PPi and ATP sensing (**D5**–**D14**) and their sensing mechanisms.

Chemosensing of phosphorous-containing anions garners large interest due to the important biological roles that these anions play in our body. Phosphates anions constitute an important buffer system in our body. Many biomolecules also carry the phosphate groups. The most important phosphorous-containing anion in our biological system would probably be adenosine triphosphate (ATP), which is responsible for providing energy at the cellular level for normal physiological functions. Known as “the molecular unit currency of intracellular energy transfer”, ATP is hydrolysed to adenosine di- and mono- phosphate (ADP and AMP) during metabolic processes, in the event, giving out energy. Orthophosphate (PO_4_^3−^, abbrev. Pi) is generated in the cell when ATP hydrolyses to ADP, whereas pyrophosphate (P_2_O_7_^4−^, abbrev. PPi) can be generated when ATP is hydrolysed into AMP. Apart from being part of the cellular-metabolic process, PPi also plays other key biological roles in the body, such as in DNA replication, calcification inhibitor in blood plasma and urine, and inhibitor of hydroxyapatite formation in extracellular fluids. An abnormal level of PPi in these biological fluids would thus be a signal of disease and health problems. In this section, recently reported AIE-based fluorescence chemosensors for the sensing of ATP, PPi and phosphates will be reviewed. The chemical structures of these chemosensors are given in Scheme 8 and Scheme 9 and their optical properties and sensing performance are summarised in Table 7.

### 5.1. Sensing of Phosphates

Phosphate anions play an important role in biological buffering systems. They include PO_4_^3−^, H_2_PO_4_^−^ and HPO_4_^−^, which are deprotonated forms of phosphoric acid H_3_PO_4_. Gao et al. reported compound **D1** with a donor-π-acceptor configuration, which possessed TICT and thereby manifest an AIE effect [103]. **D1** selectively sensed H_2_PO_4_^−^ anions via counter-anion exchange with iodide, resulting in an enhancement of blue emission in acetonitrile. Interestingly, the **D1**-H_2_PO_4_^−^ complex can be further used to sense perchlorate anions in water [103]. Wu et al. reported compound **D2** where TPE is functionalized with four bisurea groups for selective recognition and coordination with anions [101]. **D2** ligand was found to selectively sense both PO_4_^3−^ and HPO_4_^2−^ anions by forming a complex with an anion-to-ligand ratio of 4:2 (Scheme 8), thereby triggering an enhancement of blue emission (27- and 20-fold, respectively) due to RIR mechanism of TPE, i.e., anion-coordination-induced emission [101]. More recently, Zheng et al. reported selective fluorescence turn-on sensing of PO_4_^3−^ in water by TPE-derivative **D3** [102]. The emission response is due to the fact that PO_4_^3−^ abstracts a proton from **D3.2HCl** to form **D3.HCl**, which then forms an insoluble hydrogen bond network with HPO_4_^2−^ (Scheme 8) [102]. This sensing process is highly reversible, with the addition of HCl resulting in the regeneration of **D3**. Finally, a multi analyte sensor made up of AIE-doped poly(ionic liquid) **D4** was reported to sense amino acids, metal ions and phosphate derivatives found in urine samples [115].

### 5.2. Sensing of Pyrophosphates

PPi plays key biological roles and an abnormal level of PPi in biological samples may signal the presence of disease and health issues. It is therefore important to develop colorimetric or fluorescence chemosensors for detecting PPi, particularly compatible with in vivo or in vitro sensing. Scheme 9 shows the structures of some AIE-based PPi sensors (**D5**–**D10b**). The general mechanism for its detection involves coordination with PPi.

**Figure 9:**
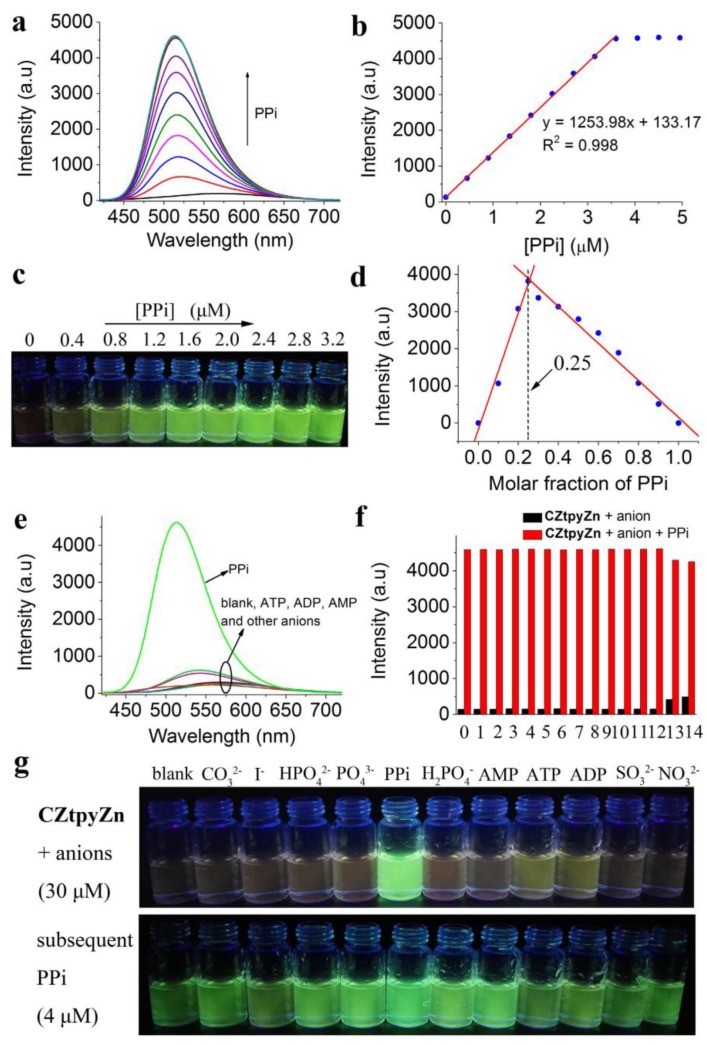
(**a**) Spectrofluorometric titration studies of **D7** with PPi, with the plot of fluorescence intensities at 515 nm vs. PPi concentration shown in (**b**). (**c**) Photos showing the emission changes of **D7** in the presence of increasing PPi concentration. (**d**) Job plot of binding study between PPi and **D7**. (**e**) Fluorescence spectra of **D7** in the separate presence of PPi and different anions. (**f**) Fluorescence responses of **D7** at 515 nm to various anions with and without PPi, with the corresponding photos shown in (**g**). Reproduced with permission from [106]. Copyright 2016, Springer Nature Publishing AG.

Sensors **D5**–**D8** contains binding sites for the selective coordination of PPi. They are often soluble as free molecular sensors in the medium of sensing, but in the process of coordinating with PPi, insoluble aggregates or self-assembly networks were formed, hence triggering the turn-on of emission. Yu et al. reported dicyclen-TPE zinc(II) complex, **D5**, as a PPi sensor where zinc(II) centres served as PPi coordination sites (Scheme 9) [104]. The addition of PPi to water-soluble **D5** resulted in 15-fold enhancement in fluorescence intensity due to the formation of poorly-soluble **D5**-PPi coordination complex, as well as RIR to the TPE moieties. Yu et al. subsequently reported two TPE-vinyl pyridinium salts, **D6a** and **D6b**, as metal-free probes of PPi [105]. It was proposed that PPi would displace iodide counteranions of **D6a** and **D6b** in an ion-exchange process, therefore leading to the formation of nanoparticles, which caused strong fluorescence enhancement (up to 45-fold increase) with blue-shifted emission wavelengths due to the ICT effect [105]. Interestingly, other organic phosphates like ATP, ADP and AMP do not trigger a similar fluorescence response. Carbazole-terpyridine zinc (II) complex **D7** sensed PPi in a similar way to **D5**. A PPi anion would coordinate to three **D7** molecules at the zinc(II) centres in a trigonal fashion (upon displacement of chloride ligands) to form insoluble (**D7**)_3_-PPi complexes which aggregated in aqueous medium [106]. This caused green fluorescence to turn on (Figure 9), due to AIE and ICT. The selective sensing of PPi over ATP, ADP and AMP, has a detection limit of 5.37 nM which is amongst the lowest for AIE-based PPi sensors [106]. Meng et al. reported a metal-free TPE-based PPi sensor, **D8**, which selectively formed self-assembly with PPi in a linear fashion via hydrogen bonding with the four peripheral -NH_2_ groups, (Scheme 9) [107]. This resulted in an increase in the fluorescence intensity of **D8** by 100-fold. The subsequent addition of alkali phosphatase (ALP), an enzyme that catalyses dephosphorylation of biomolecules, to the **D8**-PPi supramolecular network resulted in the network’s disintegration due to hydrolysis of PPi, and hence the quenching of blue emission. Therefore, **D8** was reported to sense PPi, and subsequently, ALP, with detection limits of 66.7 nM and 0.8 ng/mL, respectively [107].

The competitive coordination of PPi and metal centres can also result in displacement of their original ligand, thereby triggering an emission response of AIE-active free ligands. This strategy has been employed for AIE-based PPi sensors **D9** and **D10** (Scheme 9). Zhao et al. reported an AIE-active TPE-dipicolylamine ligand which selectively coordinated to Cu(II) to afford PPi sensor **D9 [108]**. Coordination with Cu(II) in aqueous medium effectively quenched the ligand’s emission but the subsequent addition of PPi released the ligand by competitively binding with Cu(II) to form Cu(II)-PPi complex. The free TPE-dipicolylamine ligand thereby aggregated in water resulting in the turn-on of green emission. **D9** had a low detection limit of 56 nM and could be employed for imaging of PPi in living cells [108]. Similarly, Zhu et al. reported two Eu(III)-based metallopolymers, **D10a** and **D10b**, utilizing the similar strategy for sensing PPi [109]. The polymers contain terpyridine ligands as pendants that coordinated with Eu(III). Competitive binding of PPi with Eu(III) released the free uncoordinated polymers leading to the quenching of characteristic emission of Eu^3+^ at 613 nm. Metallopolymer **D10b** was subsequently applied for the detection of PPi in living cells [109].

### 5.3. Sensing of Adenosine Triphosphates

ATP chemosensors often make use of cationic groups to bind with triply-negative charged ATP via electrostatic interactions. However, relying solely on electrostatic attraction forces may not guarantee selectivity towards ATP, especially in the presence of other organophosphates. Alternatively, labelled aptamer may be used as probes to selectively bind ATP. Nonetheless, a simpler label-free method to detect ATP is still desired and thus the need for developing simple molecular sensors for selective and sensitive sensing of ATP.

Guanidinium groups were reported to develop several AIE-based ATP sensors by utilising electrostatic interactions between positively charged guanidinium and negatively charged ATP. Earlier, Shinkai et al. reported that a TPE-derivative tethered with four peripheral guanidinium terminal groups selectively sensed ATP over ADP and AMP via an extremely large enhancement of blue fluorescence [116]. They subsequently reported another ATP-sensor **D11** which had six guanidinium-terminated arms attached to (2Z,2′Z)-2,2′-(1,4-phenylene)bis(3-phenylacrylonitrile) [110]. It was found that **D11** self-assembled with ATP in primarily aqueous medium leading to blue-green emission turn-on. Similarly, Barboiu et al. reported TPE-guanidinium derivatives **D12a** an **D12b** with greater selectivity towards ATP compared to ADP and AMP [111].

**Figure 10:**
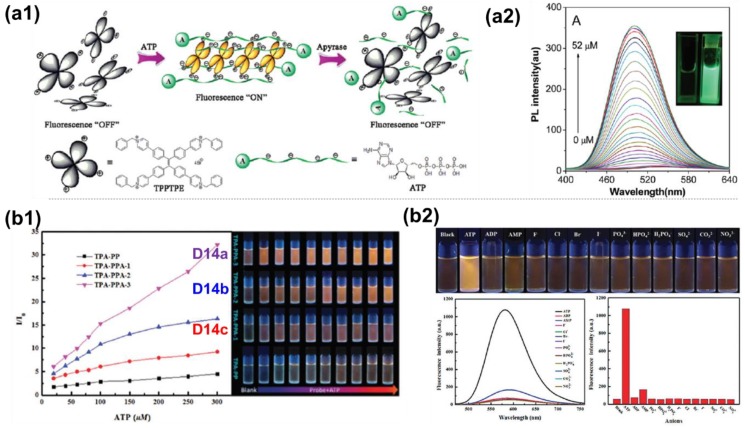
(**a1**) Schematic showing the binding of **D13** with ATP, and (**a2**) spectrofluorometric titration studies of **D13** with ATP. Insert: photos showing the turn-on of green emission of **D13** due to ATP. (**b1**) Plot of maximum emission intensities of **D14a**–**D14c** (vs. a control compound without boronic acid groups), with an increasing amount of ATP added; on the right, photos showing the corresponding emission changes. (**b2**) Fluorescence spectra and photos showing emission turn-on for **D14a** due to selective recognition of ATP. Reproduced with permission from [112,113]. Copyright 2017, the Royal Society of Chemistry.

Wang et al. reported water-soluble TPE-tetrapyridinium salt **D13** in which the four pyridinium groups could provide electrostatic attraction to bind with ATP [112]. A green emission of **D13** was turned on upon the selective binding with ATP even in the presence of ADP and AMP in HEPES buffer solution (Figure 10a). The subsequent addition of apyrase resulted in the disintegration of **D13**-ATP assembly, and hence a turn-off of fluorescence was observed [112]. Ma et al. reported a series of AIE-active triphenylamine-pyridinium compounds **D14a**, **D14b** and **D14c**, containing 1, 2 and 3 terminal phenylboronic acid groups, respectively [113]. It was proposed that pyridinium groups could bind with ATP through electrostatic attraction forces whereas boronic acids could specifically interact with *cis*-diol on the ribose moiety of ATP. Enhancement of orange emission was observed for all three sensors due to selective binding with ATP (Figure 10b) [113].

Finally, Huang et al. reported a hydrogel system that is responsive to ATP. Hydrogel **D15** was formed from the self-assembly between a TPE derivative containing two quaternary ammonium groups, and poly(sodium *p*-styrenesulfonate) [114]. Self-assembly caused RIR of the TPE moieties, leading to the hydrogel with bright blue fluorescence. When ATP was added, it competitively interacted with the two quaternary ammonium groups in the TPE derivatives via electrostatic interactions, disassembled the hydrogel and eventually quenched blue emission. The above process can however be reversed with addition of phosphatase, which hydrolyses ATP, thereby reinstating the formation of hydrogel **D15 [114]**. This type of hydrogel is biocompatible and could enter cell cytoplasm for biosensing of ATP in HEK293 cell lines.

## 6. Sensing of Nitrogen-Containing Oxoanions

**Scheme 10:**
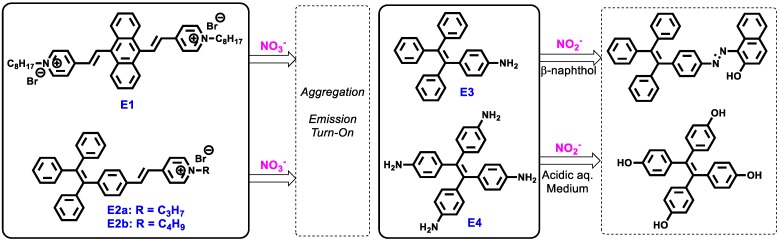
Chemical structures and mechanism of sensing of AIE-based nitrate sensors (**E1**–**E2b**) and nitrite sensors (**E3** and **E4**).

Compared to other anions, colorimetric and fluorescent chemosensors for the selective sensing of nitrogen-containing oxoanions appear relatively fewer, in spite of the prevalence and importance of these anions in industrial applications and biological systems. In this section, recent examples of AIE-based nitrate (NO_3_^−^) and nitrite (NO_2_^−^) chemosensors will be reviewed. The chemical structures of these AIE-based chemosensors are shown in Scheme 10, and their optical properties and sensing performance are summarised in Table 8.

### 6.1. Sensing of Nitrates

Nitrate anions are found naturally as mineral deposits, particularly in the form of sodium nitrate. Nitrate is well-known for its application as fertilizers due to its high water-solubility and biodegradability. The oxidizing properties of nitrates (towards carbon compounds) have also been used in gunpowder and explosives. However, nitrate toxicity and poisoning become an important health and environment problem given the widespread use of nitrate fertilizers polluting the environment and water sources. Infants, pregnant mothers and aquatic species are particularly affected by the over exposure to nitrates. Traditional ways to detect nitrate include the cadmium reduction method or the use of instrumental analysis.

**Figure 11:**
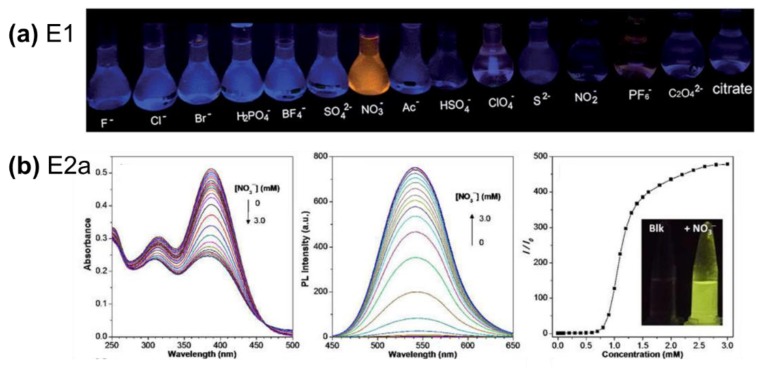
(**a**) Photos showing the selective detection of nitrate by **E1** via an observable emission turn-on. Reproduced with permission from [117]. Copyright 2015, the Royal Society of Chemistry. (**b**) Spectrophotometric and spectrofluorometric titration studies of **E2a** with nitrate, with photos showing the emission turn-on of **E2a** due to nitrate. Reproduced with permission from [65]. Copyright 2018, American Chemical Society.

AIE-based nitrate sensors **E1**, **E2a** and **E2b** (Scheme 10) structurally comprise AIE-active moieties incorporated or functionalized with pyridium groups, whereby the pyridinium groups provide ionic interactions with nitrate anions. The self-assembly of the water-soluble **E1**, **E2a** and **E2b** with nitrate formed aggregates, resulting in the emission turn-on. Ni et al. reported anthracene vinyl pyridinium salt **E1** that selectively gave rise to strong yellow emission on undergoing ionic self-assembly with nitrate in water (Figure 11a) [117]. Similarly, Zhao et al. reported TPE-vinyl pyridinium derivatives **E2a** and **E2b**, which turned on yellow emission upon the selective detection of nitrate (Figure 11b) [65]. Interestingly, **E2a** was also found to undergo similar anion-induced aggregation resulting in emission with perchlorate but turned on orange emission instead. The detection limits for **E2a** sensing of nitrate and perchlorate are 0.43 and 0.38 μM, respectively. [65]

### 6.2. Sensing of Nitrites

Unlike the nitrate anion with oxidizing capability, the nitrite anion is, however, a reducing agent. Due to its reducing property, nitrite is commonly used in the curing of meat to prevent bacterial growth. However, overconsumption of nitrite from cured meat may lead to health problems. Nitrite is considered to be toxic and carcinogenic, and the lethal dose of nitrite in human bodies is approximately 22 mg/kg of body weight. A common way of detecting nitrite in analytical samples is the Griess test, commonly used in forensic labs. In the Griess test, a deeply-coloured red azo dye would be generated from sulfanilic acid and naphthyl-1-amine in the presence of nitrite.

Diazothization reaction can be used for detection of nitrite. For example, Banerjee reported an AIE-based nitrite chemosensors in which amino-TPE (**E3**) was converted to into its diazonium salt followed by coupling with β-naphthol (Scheme 10) [118]. During this process, blue fluorescence of **E3** was quenched as the TPE-azo-naphthol compound is non-fluorescent [118]. In another example, Bhosale et al. reported that tetraamino-TPE compound **E4** showed an enhancement of blue emission in the presence of nitrite [119]. The proposed mechanism involves protonation of neutral **E4** under acidic conditions to form tetra(ammonium)-TPE, followed by diazotization of the ammonium groups (-NH_3_^+^) in the presence of nitrite into TPE-tetra(diazonium) salt. The diazonium salt would then undergo in situ hydrolysis to afford tetrahydroxy-TPE. Compared to the weakly emissive protonated-**E4** (in an acidic aqueous medium), an increase of fluorescence intensity by 20-fold was observed on the addition of nitrite anions [119].

## 7. Sensing of Other Anions

**Scheme 11:**
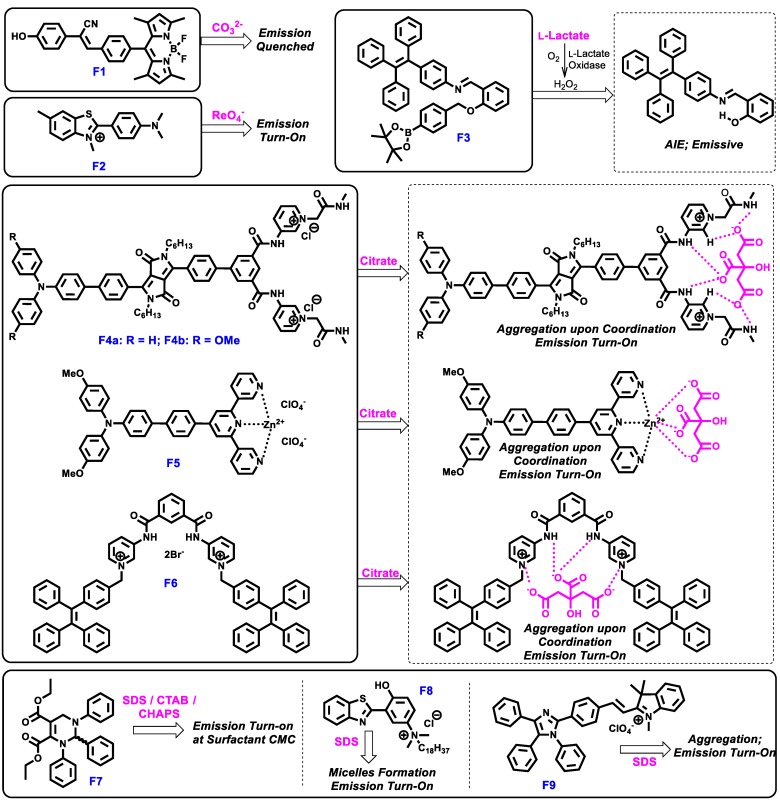
Chemical structures and detection mode of AIE-based chemosensors for the sensing of carbonate, perrhenate, lactate, citrate and anionic surfactants (**F1**–**F9**).

There are several other AIE-based chemosensors that detect less common anionic species. In this section, chemosensors for the sensing of carbonate, perrhenate, lactate, citrate and anionic surfactants will be highlighted. The chemical structures and modes of detection of these AIE-based sensors are illustrated in Scheme 11, and their optical properties and sensing performances are summarised in Table 9.

### 7.1. Sensing of Carbonates

Optical carbonate (CO_3_^2^^−^) chemosensors are rare. One recent example of AIE-based carbonate sensors involved AIE-active cyano-diphenylethylene-conjugated BODPY, **F1** [120]. Containing a hydroxy group in cyanodiphenylethylene, **F1** appeared to be pH-sensitive (p*K*_a_ = 9.79), exhibiting bright green emission at neutral pH but non-emissive under basic conditions, due to the hydroxy deprotonation that was readily converted the cyanodiphenylethylene group to a quinoid form. Making use of this pH-sensitivity, **F1** was able to sense carbonate anions (p*K*_a_ = 10.3) in water whereby green emission of **F1** would be quenched [120].

### 7.2. Sensing of Perrhenate

The contamination of the environment and water sources by radioactive materials is a pertinent issue in some countries, which poses a health and safety threat. One of the highly toxic radioactive oxoanion contaminants of interest is pertechnetate (^99^TcO_4_^−^). Due to its danger, access to pertechnetate for research purpose is unfortunately restricted, which prompted Singh et al. to devise a chemosensor for the detection of a chemically-similar but non-radioactive oxoanion, perrhenate (ReO_4_^−^), to mimic potential environment-sensing of pertechnetate. Singh et al. reported thioflavin-T (**F2**) to sense perrhenate via the formation of contact ion pairs, which resulted in aggregation and hence the turning-on of emission [121]. An enhancement of emission peaking at 520 nm indeed arose from the aggregation of **F2** with perrhenate in water, which gave a detection limit of 260 μM.

### 7.3. Sensing of L-Lactate

L-lactate is produced by skeletal muscles and brain tissues during anaerobic respiration to supply energy for basic metabolism activities. L-lactate is removed by the kidney and liver to maintain body pH, and any excessive accumulation of L-lactate will signal severe health issues present. Therefore, the sensing of L-lactate, an important biomarker, is crucial. Ng et al. developed a sensitive and selective detection method for sensing L-lactate involving TPE-boronic ester derivative **F3** and L-lactate oxidase (LOx), for a duration of 20–60 min [121]. LOx is an enzyme that converts L-lactate to pyruvate in the presence of oxygen. In the process, hydrogen peroxide is given out, which cleaves the methylene phenylboronic ester group to form an emissive TPE derivative in its aggregation state in aqueous solution. (Scheme 11) [122].

### 7.4. Sensing of Citrates

Being part of the Krebs cycle that produces ATP from the aerobic metabolism of glucose, citrate plays an important biological role in our body. In addition, monitoring citrate levels in biological fluids can signal the presence of any health issues. For example, the possibility of kidney dysfunction can be inferred from the lowering of citrate level in urine samples. Conventional means of monitoring a citrate level involves instrumental analysis. Nonetheless, the development of optical ratiometric sensors for sensitive detection of citrate could pave the way for cheaper, more affordable, faster and more convenient means of clinical testing. Citrate anion consists of three carboxylate groups (-COO^−^) and a tertiary alcohol (-OH) group. Therefore, it would be logical for citrate-selective sensors to involve coordination and anion-recognition of citrate. Generally, the coordination of citrate at binding sites of these citrate sensors would impose RIR to AIE-active moieties, and/or lead to aggregation, which would result in the effective turn-on of emission.

**Figure 12:**
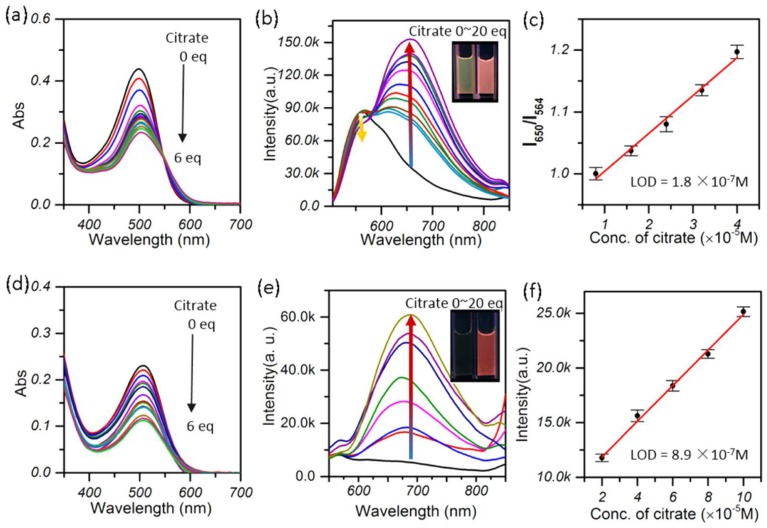
Spectrophotometric titration of **F4a** (**a**) and **F4b** (**d**); and spectrofluorometric titration of **F4a** (**b**) and **F4b** (**e**) with increasing amount of citrate. Inserts in (**b**) and (**e**): photos showing emission changes to the two sensors in response to citrate. The plot of fluorescence intensities as a function of citrate concentration for **F4a** and **F4b** are shown in (**c**) and (**f**), respectively. Reproduced with permission from [123]. Copyright 2016, the American Chemical Society.

Hua et al. reported two triphenylamine-diketopyrrolopyrrole (DPP)-based molecular sensors, **F4a** and **F4b,** containing pyridinium-based symmetrical diamides as a recognition group for citrate anions [123]. Coordination of citrate at the recognition sites of **F4a** and **F4b** via a series of hydrogen bonding interactions would result in aggregation and thereby the turning-on of red emission (Figure 12). Hua et al. also simultaneously reported triphenylamine-terpyridine Zn(II) complex **F5** which coordinated with citrate at the Zn(II) centre, leading to the weakening of fluorescence-quenching effect of Zn(II) on triphenylamine-terpyridine ligand, as well as the aggregation of **F5**-citrate complex, hence lighting up of yellowish-green fluorescence [124]. More recently, Hua et al. reported a TPE-derivative, **F6**, which contained bis(pyridinium) isophthalamide as a citrate recognition receptor [125]. The formation of nanoparticles upon coordination of **F6** with citrate led to significant enhancement of blue emission, attributing to RIR of TPE. All three AIE-based citrate sensors exhibited a considerably consistent and low detection limit in the 0.1 μM range.

### 7.5. Sensing of Anionic Surfactants

Anionic surfactants are commonly used in pharmaceutical and consumer-care products, in particular, soap and detergents. While they serve their function in reducing interfacial tensions and thus improve solubility of active ingredients, dirt or grease, the discharge of them into water source can result in pollution that harms aquatic wildlife. Surfactants are known to form micelles and aggregates. Therefore, the incorporation of AIE-active moieties into chemosensor design can be used as a sensing strategy in the developing of surfactant chemosensors.

Liu et al. reported the use of AIE-active compound **F7**, to determine the critical micelle concentration (CMC) of three surfactants via a blue-emission turn-on: anionic sodium dodecyl sulfate (SDS), zwitterionic 3-[(3-cholamidopropyl)dimethylammonio]-1-propanesulfonate (CHAPS), and cationic cetrimonium bromide [126]. Tang et al. reported using 2-(2-hydroxyphenyl)benzothiazole (HBT) derivative, **F8**, to sense SDS via aggregates and/or micelles formation [127]. Cationic **F8** and anionic SDS would interact via synergistic electrostatic and hydrophobic interactions, leading to the turn-on of green-emission [127]. Similarly, using the same mechanism, the aggregation of AIE-active and water-soluble **F8**, in the presence of SDS, resulted in the turn-on of red emission due to RIR, making it a good anionic surfactant sensor with a low detection limit of 48 nM [128].

## 8. Conclusions and Perspective

This review summarised recent development of AIE-based luminescent chemosensors for anion detection. The examples showcased herein were categorised according to different classes of anions: (1) halides and halogen-containing anions, (2) cyanide, (3) sulphur-containing oxoanions, (4) phosphorus-containing anions, (5) nitrogen-containing oxoanions, and (6) other miscellaneous anions including citrate and anionic surfactants. In terms of chemical characteristics of different anions such as nucleophilicity, basicity/acidity, electrochemistry and coordination ability, a variety of AIE-based chemosensors with high anion selectivity have been designed and prepared by incorporation of AIE-active moieties in the chemosensor molecules, allowing for the use of its solubility, segregation, aggregation, assembly and distinct interactions with anionic analytes to monitor changes in fluorescence. This has further extended to the effective and sensitive detection of analytes in aqueous medium, which is particularly important for sensing in biological samples, as well as the detection of water contamination.

Detection mechanisms are highly dependent on the nature of the individual anions. For instance, the sensing of fluorides, cyanides and sulphites in general involves nucleophilic reactions with electron-deficient sensing groups in chemosensor molecules. Fluorides and cyanides are able to deprotonate acidic -OH protons of chemosensor molecules, leading to further chemical changes or triggering an optical response. The sensing of bisulphites often requires Michael acceptors to undergo Michael addition reaction with the targeted anion. Sensing of oxidative hypochlorite anions can be achieved by undergoing oxidative-type reactions, e.g., oxidative cleavage of C=C bonds, whereas nitrite chemosensors can make use of diazotization of the amino groups. In contrast, some chemosensors function based on electrostatic interactions, hydrogen bonding and anionic recognition by specific receptors. These can be specifically applied to less reactive anions like chlorides, bisulphates, phosphates, pyrophosphates, ATP, nitrates, citrates and anionic surfactants. Amongst these anions, pyrophosphates and citrates exist as multidentate ligands with multiple coordination or hydrogen bonding sites, and thus detection often involves specific binding with anionic receptors. The coordination or electrostatic interactions between anions and chemosensors frequently results in the formation of insoluble aggregates, supramolecular self-assembly networks, or even micelles, which can impose RIM on the AIE-active moieties, hence triggering the turn-on of emission. Alternatively, the preferential and competitive coordination of anions with certain cations or cationic groups in the chemosensors can lead to disruption or disintegration of the existing AIE supramolecular network (e.g., hydrogels), thereby resulting in emission changes, such as change in emission colour or the quenching of emission.

Moving forward, there are still many challenges that need to be addressed in this field. Firstly, anion selectivity can be further improved. For instance, there are some common sensing groups, like dicyanovinyl, which are reactive with more than one nucleophilic anion. Secondly, compared to fluorides and cyanides, effective detection of some anions, such as nitrogen-containing oxoanions, sulphur-containing oxoanions, chlorides and bromides, remains scarce. Thirdly, it would be of interest if AIE-based fluorescent chemosensors can be developed to selectively sense multiple anions with different responses, e.g., colour changes for each different anion. A multiple-analyte sensor array that is made up of single or multiple chemosensors could then be developed with different responses providing simultaneous sensing of different anions and cations present [129]. Finally, it would be envisaged for these intelligently-developed chemosensors to be translated into commercial applications, therefore fulfilling their potential as low-cost, easy- and convenient-to-use chemosensors for important practical applications of biological and environmental sensing.

## Figures and Tables

**Table 1:** Summary of optical properties and sensing performance of AIE-based fluoride chemosensors **A1**–**A13**.

	λ_abs_ (nm) ^a^	λ_em_ (nm) ^b^	Φ (%) ^c^	Original Emission Colour	Emission Colour/Change in the Presence of Anion	L.O.D ^d^	Mechanism of Detection	Ref.
**A1**	280, 329, 394	486 (soln) ^e^; ca. 525 (aggr) ^f^	-	Green/Blue (aggr) ^f^	Quenched	1.02 μM	Reaction with boron centre	[43]
**A2**	434	481	9 (soln) ^e^; 17 (aggr) ^f^	Green (in PMMA)	Quenched	1.52 ppm	Reaction with boron centre	[44]
**A3**	468	567 (soln) ^e^ 590 (aggr) ^f^	1.5 (soln) ^e^; 5.6 (aggr) ^f^	Yellow (in PMMA)	Partially quenched	2.82 ppm	Reaction with boron centre	[44]
**A4**	368	409 (soln) ^e^; 481 (film) ^g^	1 (soln) ^e^; 64 (film)	Blue (aggr) ^f^; Partially	Quenched	-	Reaction with boron centre	[45]
**A5**	495	524 (soln) ^e^; 540 (aggr) ^f^	21 (soln) ^e^	Green (aggr) ^f^	Weak teal	0.345 μM	Reaction with boron centre	[46]
**A6**	305, 350	530 (soln) ^e^; 559 (solid) ^g^	0.1 (soln) ^e^; 19 (solid) ^g^	Yellow (aggr; solid) ^f,g^	Bright green	14.0 pM	Hydrogen bonding of Schiff Base’s OH with F^−^ followed by deprotonation	[47]
**A7**	~388	536 (aggr) ^f^; 508 (+F^−^) ^h^	-	-	Green, turn-on	90 nM	F^−^-mediated cleavage of triisopropylsily group	[48]
**A8**	~315	470	-	-	Blue, turn-on	3.8 nM	F^−^-mediated cleavage of thiophosphinate group leading to AIE	[49]
**A9**	~370	550 (soln) ^e^ 470 (+F^−^) ^h^	-	Orange (aggr) ^f^	Green	0.6 mM	Displacement of I^−^ counter-anion leading to twisted conformation	[50]
**A10**	340 (+F^−^) ^h^	430 (+F^−^) ^h^	-	-	Blue; enhanced	0.202 μM	Aggregation with F^−^	[51]
**A11**	-	430	-	-	Blue; turn-on	8.67 nM	Supramolecular assembly with F^−^	[52]
**A12**	-	468 (+Al^3+^) ^h^	-	Blue (+Al^3+^) ^h^	Quenched	0.6 μM	Regeneration of **A12** from its complex with Al^3+^ via forming AlF_3_	[53]
**A13**	-	410	-	Blue (Gel) ^g^; Brilliant-Blue-	Green (Gel + Zn^2+^ + F^−^) ^g,h^; greenish yellow (Gel + F^−^) ^g,h^	1.3 μM	Changes in supramolecular interactions involved in gelation of **A13**	[54]

^a^ Absorption maxima; ^b^ Emission maxima; ^c^ Fluorescence quantum yield; ^d^ Limit of Detection; ^e^ (soln) denotes optical properties in solution state; ^f^ (aggr) denotes optical properties of aggregates; ^g^ (solid/film/gel) denotes optical properties in solid/film/gel state; ^h^ (+X^n+/−^) denotes optical properties in the presence of ion X^n+/−^. “-”: data were not reported.

**Table 2:** Summary of optical properties and sensing performance of AIE-based chloride chemosensors **A14**–**A15**.

	λ_abs_ (nm) ^a^	λ_em_ (nm) ^b^	Φ (%) ^c^	Original Emission Colour	Emission Colour/Change in the Presence of Anion	L.O.D ^d^	Mechanism of Detection	Ref.
**A14**	-	515 (aggr, + Cl^−^) ^e,g^	-	-	Bright green, turn-on	-	Coordination with Cl^−^	[58]
**A15**	-	460 (Gel) ^f^; 445 (Gel + Cl^−^) ^f,g^	-	Blue (gel) ^f^	Quenched	24.9 μM	Degradation of supramolecular polymeric network	[59]

^a^ Absorption maxima; ^b^ Emission maxima; ^c^ Fluorescence quantum yield; ^d^ Limit of Detection; ^e^ (aggr) denotes optical properties of aggregates; ^f^ (solid/film/gel) denotes optical properties in solid/film/gel state; ^g^ (+X^n+/−^) denotes optical properties in the presence of ion X^n+/−^. “-”: data were not reported.

**Table 3:** Summary of optical properties and sensing performance of AIE-based hypochlorite chemosensors **A16**–**A19**, and perchlorate chemosensor **A20** and **E2a**.

	λ_abs_ (nm) ^a^	λ_em_ (nm) ^b^	Φ (%) ^c^	Original Emission Colour	Emission Colour/Change in the Presence of Anion	L.O.D ^d^	Mechanism of Detection	Ref.
*Hypochlorite (ClO* ^−^ *) Sensors*								
**A16**	250, 321	470 (aggr) ^f^	1.0 (soln) ^e^; 33.0 (aggr) ^f^	Sky blue (aggr) ^f^	Quenched	28 nM	Boronic ester groups converted to –OH groups, then **A16** further oxidised to water-soluble quinone form	[60]
**A17**	~340, 420	~595 (aggr) ^f^	-	Strong red FL (aggr) ^f^	Weak green	0.47 μM	1,1′-dicyanovinyl group converted to aldehyde group	[61]
**A18**	538	514 (aggr, +ClO^−^) ^f,h^	15.7 (+ClO^−^) ^h^	-	Blue-green, turn-on	13.2 nM	Cleavage of C=C bond into aldehyde group, detaching the indole group that causes TICT.	[62]
**A19**	-	469 (aggr) ^f^	1.1; 6.7(+ClO^−^) ^h^	Blue (aggr) ^f^;	Blue, enhanced	75 nM	Cleavage of C=C bond carrying 2-thioxo-dihydro-pyrimidine-4,6-dione groups.	[63]
*Perchlorate (ClO_4_* ^−^ *) Sensors*								
**A20**	~375	650 (soln) ^e^; 632 (aggr, +ClO_4_^−^) ^f,h^	-	-	Red, turn-on	0.96 μM	ClO_4_^−^-mediated aggregation	[64]
**E2a**	396	580 (soln) ^e^; 570 (aggr) ^f^; 575 (solid) ^g^	14.2 (soln) ^e^; 2.1 (aggr) ^f^; 35.9 (solid) ^g^	Red (soln) ^e^	Orange	0.38 μM	ClO_4_^−^ induced self-assembly/aggregation	[65]

^a^ Absorption maxima; ^b^ Emission maxima; ^c^ Fluorescence quantum yield; ^d^ Limit of Detection; ^e^ (soln) denotes optical properties in solution state; ^f^ (aggr) denotes optical properties of aggregates; ^g^ (solid/film/gel) denotes optical properties in solid/film/gel state; ^h^ (+X^n+/−^) denotes optical properties in the presence of ion X^n+/−^. “-”: data were not reported.

**Table 4:** Summary of optical properties and sensing performance of AIE-based iodide chemosensors **A21**–**A23**.

	λ_abs_ (nm) ^a^	λ_em_ (nm) ^b^	Φ (%) ^c^	Original Emission Colour	Emission Colour/Change in the Presence of Anion	L.O.D ^d^	Mechanism of Detection	Ref.
**A21**	~310, 365 (solid) ^f^	490 (soln) ^e^; 530 (solid) ^f^	45 (soln) ^e^; 89 (solid) ^f^	Green (solid) ^f^	Quenched	-	Emission quenched due to ionic interaction between Ag^+^ and I^−^	[66]
**A22**	-	~525	-	Blue	Quenched	0.1 μM	Emission quenched due to the release and coordination of Pb^2+^ with I^−^	[67]
**A23**	-	410	-	Blue	Quenched	94 nM	Emission quenched due to changes in supramolecular interactions involving I^−^	[68]

^a^ Absorption maxima; ^b^ Emission maxima; ^c^ Fluorescence quantum yield; ^d^ Limit of Detection; ^e^ (soln) denotes optical properties in solution state; ^f^ (solid/film/gel) denotes optical properties in solid/film/gel state; “-”: data were not reported.

**Table 5:** Summary of optical properties and sensing performance of AIE-based cyanide chemosensors **B1a**–**B14**.

	λ_abs_ (nm) ^a^	λ_em_ (nm) ^b^	Φ (%) ^c^	Original Emission Colour	Emission Colour/Change in the Presence of Anion	L.O.D ^d^	Mechanism of Detection	Ref.
**B1a**	~400 (aggr) ^f^	547 (aggr) ^f^	-	Green-yellow (aggr) ^f^	Weak blue	63 ppm	Nucleophilic addition of CN^−^ to dicyanovinyl- group	[73]
**B1b**	~400 (aggr) ^f^	565 (aggr) ^f^	-	Orange (aggr) ^f^	Weak blue	109 ppm	Nucleophilic addition of CN^−^ to dicyanovinyl- group	[73]
**B2a**	387–407 (soln) ^e^	628 (soln) ^e^; 600 (aggr) ^f^	24 (Solid) ^g^	Orange (aggr) ^f^	Quenched	0.32 μM	Nucleophilic addition of CN^−^ to dicyanovinyl- group	[74]
**B2b**	385–404 (soln) ^e^	625 (soln) ^e^; 600 (aggr) ^f^	22 (Solid) ^g^	Red (soln) ^e^; orange (aggr) ^f^	Quenched	0.29 μM	Nucleophilic addition of CN^−^ to dicyanovinyl- group	[74]
**B3a**	437 (soln) ^e^; 465 (aggr) ^f^	568 (aggr) ^f^	-	Orange (aggr) ^f^	Quenched	9.88 nM	Nucleophilic addition of CN^−^ to dicyanovinyl- group	[75]
**B3b**	440	650 (aggr) ^f^	-	Red (aggr) ^f^	Quenched	0.11 nM	Nucleophilic addition of CN^−^ to dicyanovinyl- group	[76]
**B4**	340, 390, 460 (soln) ^e^	605 (soln) ^e^	-	Orange (soln) ^e^	Quenched	-	Nucleophilic addition of CN^−^ to dicyanovinyl- group	[77]
**B5**	~450 (aggr) ^f^	580 (aggr) ^f^	-	Orange (aggr) ^f^	Quenched	0.22 μM	Nucleophilic addition of CN^−^ to cyanoethylene- group	[78]
**B6a**	562 (soln) ^e^	-	-	Non emissive (aggr; solid) ^f,g^	Blue	24.5 μM	Nucleophilic addition of CN^−^ to 1,1-dicyanomethylidene group	[79]
**B6b**	443 (soln) ^e^	629 (aggr) ^f^; 653 (solid) ^g^	-	Weak red (aggr, solid) ^f,g^	-	1.7 μM	Nucleophilic addition of CN^−^ to 1,1-dicyanomethylidene group	[79]
**B6c**	386 (soln) ^e^	573 (soln) ^e^; 543 (solid) ^g^	0.4 (soln) ^e^; 3.0 (aggr) ^f^	Yellow (aggr; solid) ^f,g^	Blue	5.07 μM	Nucleophilic addition of CN^−^ to 1,1-dicyanomethylidene group	[79]
**B7**	380	435	-	Blue	Enhanced	55 nM	Nucleophilic attack of CN^−^ to indolium moiety disrupts ICT effect	[80]
**B8**	434 (soln) ^e^	547 (soln) ^e^; 623 (aggr) ^f^	-	Green (soln) ^e^; orange (aggr) ^f^	Quenched	67.4 nM	Nucleophilic attack of CN^−^ to C=C bond interrupting ICT between carbazole and barbituric acid	[81]
**B9**	356 (soln) ^e^	n.d. (soln; aggr) ^e,f^; 464 (soln, +CN^−^) ^e,h^; 508 (aggr, +CN^−^) ^f,h^	36.6 (soln, +CN^−^) ^e,h^	No emission	Blue (soln) ^e^ green (aggr) ^f^	0.592 μM	Underwent oxidative cyclization reaction after deprotonation of −OH by CN^−^ to generate hydroxyphenyl-benzoxazole	[82]
**B10**	396, 411 (soln) ^e^	530 (soln) ^e^; 584 (aggr) ^f^	0.21 (solid) ^g^	Green (aggr) ^f^	Yellow	0.81 μM	Deprotonation of OH groups by CN^−^ leading to ICT effect that red-shifts fluorescence	[83]
**B11**	410 (soln) ^e^	516 (Gel); 498 (Gel, +CN^−^) ^g,h^	-	Green (gel) ^g^	Blue (gel) ^g^	3.02 μM	Deprotonation of OH and NH groups by CN^−^ disrupts hydrogen bonding in self-assembly	[84]
**B12**	-	410 (aggr) ^f^	11.24 (soln) ^e^; 37.8 (aggr) ^f^	Blue (aggr) ^f^; quenched (+Hg^2+^) ^h^	Blue FL recovered	77.1 nM	CN^−^ reinstates self-assembly of **B12** by removing Hg^2+^ in **B12**-Hg adduct as Hg(CN)_2_	[85]
**B13**	~345	435 (aggr) ^f^	-	Blue (aggr) ^f^; quenched (+picric acid) ^h^	Blue FL recovered	0.745 μM	CN^−^ reinstates self-assembly of **B13** by causing **B13**-Picric acid adduct to break apart	[86]
**B14**	313	498 (soln) ^e^; 458 (aggr) ^f^	3.9 (soln) ^e^; 24 (aggr) ^f^	-	Blue, turn-on	0.59 μM	Coordination of CN^−^ to Co(II) centre decreases complex solubility	[87]

^a^ Absorption maxima; ^b^ Emission maxima; ^c^ Fluorescence quantum yield; ^d^ Limit of Detection; ^e^ (soln) denotes optical properties in solution state; ^f^ (aggr) denotes optical properties of aggregates; ^g^ (solid/film/gel) denotes optical properties in solid/film/gel state; ^h^ (+X^n+/−^) denotes optical properties in the presence of ion X^n+/−^. “-”: data were not reported.

**Table 6:** Summary of optical properties and sensing performance of AIE-based bisulphite, sulphite and bisulphate chemosensors **C1**–**C5**.

	λ_abs_ (nm) ^a^	λ_em_ (nm) ^b^	Φ (%) ^c^	Original Emission Colour	Emission Colour/Change in the Presence of Anion	L.O.D ^d^	Mechanism of Detection	Ref.
*Bisulphite (HSO_3_* ^−^ *) Sensors*								
**C1**	480 (soln) ^e^; ~460, 562 (aggr) ^f^	615 (soln) ^e^; 467 (aggr) ^f^	69.3 (aggr) ^f^	Red (soln) ^e^; Green (aggr) ^f^	Blue	89 nM	Nucleophilic addition to C=C linkage effectively cuts off ICT between pyrene and benzthiazolium unit.	[89]
**C2**	390 (soln) ^e^; 435 (aggr) ^f^	440 (soln) ^e^; 460 (aggr) ^f^;	7.8 (DMSO); 2.9 (MeCN); 19.9 (aggr) ^f^	Yellow	Quenched	1.9 nM	Nucleophilic addition to C=C linkage effectively cuts off ICT between pyrene and pyridine unit.	[90]
**C3**	436	504 (aggr, +HSO_3_^−^) ^e,h^	4.52 (crystal) ^g^	Red	Green, Turn-on	1.04 ppm	Nucleophilic addition to C=C bond of aqueous-soluble sensor produces insoluble AIE product.	[91]
*Sulphite (SO*_3_^−^ *) Sensors*								
**C4**	402 (soln) ^e^; 420 (aggr) ^f^	616 (soln) ^e^; 570 (aggr) ^f^	1.9 (soln) ^e^	Yellow (aggr; solid) ^f,g^	Weak blue	7.4 nM	Nucleophilic addition of SO_3_^2−^ to dicyanovinyl group	[92]
**B3a**	437 (soln) ^e^; 465 (aggr) ^f^	568 (aggr) ^f^	-	Orange (aggr) ^f^	Quenched	0.107 μM	Nucleophilic addition of SO_3_^2−^ to dicyanovinyl group	[75]
*Bisulphate (HSO*_4_^−^ *) Sensor*								
**C5**	-	482	5.78	Blue	Enhanced	-	Selective recognition of HSO_4_^−^ guest within ion-pair receptor C5 at imidazole binding sites	[93]

^a^ Absorption maxima; ^b^ Emission maxima; ^c^ Fluorescence quantum yield; ^d^ Limit of Detection; ^e^ (soln) denotes optical properties in solution state; ^f^ (aggr) denotes optical properties of aggregates; ^g^ (solid/film/gel) denotes optical properties in solid/film/crystal state; ^h^ (+X^n+/−^) denotes optical properties in the presence of ion X^n+/−^. “-”: data were not reported.

**Table 7:** Summary of optical properties and sensing performance of AIE-based sensors for the sensing of phosphorous-containing anions (**D1**–**D14**).

	λ_abs_ (nm) ^a^	λ_em_ (nm) ^b^	Φ (%) ^c^	Original Emission Colour	Emission Colour/Change in the Presence of Anion	L.O.D ^d^	Mechanism of Detection	Ref.
*Phosphate (PO*_4_^3^^−^ *; HPO*_4_^2^^−^ *) Sensors*								
**D1**	340	454	-	Blue	Enhanced	-	Anion exchange and complex formation.	[103]
**D2**	-	500 (aggr) ^f^; 494 (+PO_4_^3−^; + HPO_4_^2−^) ^h^	9.1; 41.3 (+PO_4_^3−^) ^h^	Blue	Enhanced	-	Anion-coordination induced emission	[101]
**D3**	257, 310	493 (aggr) ^f^; 502 (aq., +PO_4_^3−^) ^e,h^	-	Blue-green (aggr) ^f^	Turn-on	-	Aqueous soluble dihydrogen chloride salt transformed to less soluble mono-hydrogen chloride salt leading to aggregation.	[102]
*Pyrophosphate (PPi) Sensors*								
**D5**	~350 (aq.) ^e^	468 (aq.) ^e^	0.426 (aq.) ^e^; 1.757 (+PPi) ^h^	-	Blue, turn-on	22.8 nM	Coordination of Zn(II) centres with PPi leading to poorer solubility and aggregation of **D2**-PPi complexes	[104]
**D6a**	~370	625 (soln)^e^; 531 (aq., +PPi) ^e,h^	0.136 (aq.) ^e^; 3.283 (+PPi) ^h^	-	Green-yellow, turn-on	133 nM	PPi replaces iodide counteranion and forms nanoparticles via supramolecular self-assembly.	[105]
**D6b**	~380	625 (soln) ^e^; 562 (aq., +PPi) ^e,h^	0.127 (aq.) ^e^, 4.143 (+PPi) ^h^	-	Orange, turn-on	118 nM	PPi replaces iodide counteranion and forms nanoparticles via supramolecular self-assembly.	[105]
**D7**	285, 316, 387	570 (aq.) ^e^; 515 (+PPi) ^h^	-	-	Green, turn-on	5.37 nM	Coordination of Zn(II) centre with PPi leading to poorer solubility and aggregation of **D4**-PPi complexes.	[106]
**D8**	-	470 (aggr; aq., +PPi) ^e,f,h^	0.5 (aq.) ^e^; 19 (aq., +PPi) ^e,h^	Blue emission (aggr) ^f^	Blue, turn on	66.7 nM	Supramolecular self-assembly of PPi and **D5** via hydrogen bonding leading to poorer solubility and aggregation	[107]
**D9**	350	500 (+PPi) ^h^	-	No emission	Green, turn-on	56 nM	Competitive coordination of PPi with Cu^2+^ releases AIE-active TPE-dipicolylamine ligand	[108]
**D10a**	400	420, 613	-	Blue (soln) ^e^	Pink; quenching of Eu^3+^’s 613nm emission	~200 nM	Competitive coordination of PPi with Eu^3+^ releases terpyridine-polymeric ligand	[109]
**D10b**	400	450, 613	-	Blue (soln) ^e^	Pink; quenching of Eu^3+^’s 613nm emission	12 nM	Competitive coordination of PPi with Eu^3+^ releases terpyridine-polymeric ligand	[109]
*ATP Sensors*								
**D11**	371	514	-	-	Blue-green, turn on	6 μM	Guanidinium-ATP binding via electrostatic interactions led to aggregation of adduct.	[110]
**D12a**	330	516 (+ATP)	-	-	Green, turn-on	-	Guanidinium-ATP binding via electrostatic interactions led to aggregation of adduct.	[111]
**D12b**	330	516 (+ATP)	-	-	Green, turn-on	-	Guanidinium-ATP binding via electrostatic interactions led to aggregation of adduct.	[111]
**D13**	~370	~535 (soln) ^e^; ~575 (aggr) ^f^; 500 (+ATP)	-	Weak orange (soln) ^e^; Yellow (aggr) ^f^	Intense green	75 nM	Binding and aggregation of **D13** and ATP via electrostatic interactions and hydrophobic interactions	[112]
**D14a**	432	609 (THF soln) ^e^	14.23 (solid) ^g^	Yellow (solid) ^g^	Orange, enhanced	~20 ppm	Specific binding and aggregation of **D14a** and ATP	[113]
**D14b**	437	614 (THF soln) ^e^	8.57 (solid) ^g^	Orange (solid) ^g^	Orange, enhanced	~3 ppm	Specific binding and aggregation of **D14b** and ATP	[113]
**D14c**	451	614 (THF soln) ^e^	13.86 (solid) ^g^	Orange (solid) ^g^	Orange, enhanced	0.3 ppm	Specific binding and aggregation of **D14c** and ATP	[113]
**D15**	-	~480	-	Blue (gel) ^g^	Quenched	-	Disintegration of self-assembled AIE hydrogel **D15**	[114]

^a^ Absorption maxima; ^b^ Emission maxima; ^c^ Fluorescence quantum yield; ^d^ Limit of Detection; ^e^ (soln/aq.) denotes optical properties in solution/aqueous state; ^f^ (aggr) denotes optical properties of aggregates; ^g^ (solid/film/gel) denotes optical properties in solid/film/gel state; ^h^ (+X^n+/−^) denotes optical properties in the presence of ion X^n+/−^. “-”: data were not reported.

**Table 8:** Summary of optical properties and sensing performance of AIE-based nitrate sensors (**E1**–**E2b**) and nitrite sensors (**E3** and **E4**).

	λ_abs_ (nm) ^a^	λ_em_ (nm) ^b^	Φ (%) ^c^	Original Emission Colour	Emission Colour/Change in the Presence of Anion	L.O.D ^d^	Mechanism of Detection	Ref.
*Nitrate (NO*_3_^−^ *) Sensors*								
**E1**	-	626 (crystal) ^g^; ~600 (aggr) ^f^; 625 (soln) ^e^	-	Strong Yellow (aggr) ^f^	Yellow, turn-on	0.475 μM	Ionic self-assembly with NO_3_^−^ in water	[117]
**E2a**	396	580 (soln) ^e^; 570 (aggr) ^f^; 575 (solid) ^g^	14.2 (soln) ^e^; 2.1 (aggr) ^f^; 35.9 (solid) ^g^	Red (soln) ^e^;	Bright yellow	0.43 μM	NO_3_^−^ induced self-assembly/aggregation	[65]
**E2b**	396	580 (soln) ^e^; 570 (aggr) ^f^; 560 (solid) ^g^	13.8 (soln) ^e^; 2.0 (aggr) ^f^; 34.7 (solid) ^g^	Red (soln) ^e^;	Bright yellow	-	NO_3_^−^ induced self-assembly/aggregation	[65]
*Nitrite (NO*_2_^−^ *) Sensors*								
**E3**	~345	447 (aggr) ^f^	-	Blue (aggr) ^f^	Quenched	0.6 μM	Diazotization of amino group in the presence of NO_2_^−^ followed by coupling with naphthol to form non-fluorescent coupled-product	[118]
**E4**	273, 345 (soln) ^e^	510 (+H^+^) ^h^	-	Weak blue (+H^+^) ^h^	Enhanced	17.7 ppb	Diazotization reaction resulting in the conversion of amino groups to hydroxyl groups.	[119]

^a^ Absorption maxima; ^b^ Emission maxima; ^c^ Fluorescence quantum yield; ^d^ Limit of Detection; ^e^ (soln) denotes optical properties in solution state; ^f^ (aggr) denotes optical properties of aggregates; ^g^ (solid/film/gel/crystal) denotes optical properties in solid/film/gel/crystal state; ^h^ (+X^n+/−^) denotes optical properties in the presence of ion X^n+/−^. “-”: data were not reported.

**Table 9:** Optical properties and sensing performances of AIE-based chemosensors for the sensing of carbonate, perrhenate, lactate, citrate and anionic surfactants (**F1**–**F9**).

	λ_abs_ (nm) ^a^	λ_em_ (nm) ^b^	Φ (%) ^c^	Original Emission Colour	Emission Colour/Change in the Presence of Anion	L.O.D ^d^	Mechanism of Detection	Ref.
*Carbonate (CO*_3_^2−^ *) Sensor*								
**F1**	361, 505 (soln) ^e^	370, 528 (soln) ^e^	-	Green (aggr) ^f^	Quenched	40 μM	pH sensitivity of phenol group with pKa value close to that of CO_3_^2−^	[120]
*Perrhenate (ReO*_4_^−^ *) Sensor*								
**F2**	412	490 (aq.) ^e^; 520 (+ReO_4_^−^) ^h^	-	-	Turn-on	260 μM	ReO_4_^−^ forms contact ion pair with cationic **F2** leading to aggregation hence AIE	[121]
*L-Lactate Sensor*								
**F3**	373	530	-	-	Green, turn-on	5.5 μM	H_2_O_2_ converted from L-Lactate in the presence of LOx oxidises methylene phenyl boronic ester group of **F3** to generate emissive AIE product.	[122]
*Citrate Sensors*								
**F4a**	500	564 (soln) ^e^; 650 (aggr) ^f^	-	Yellow-green (soln) ^e^	Red, turn-on	0.18 μM	Aggregation of **F4a** upon coordination with citrate leading to emission turn-on due to AIE	[123]
**F4b**	505	672 (aggr) ^f^	-	-	Red, turn-on	0.89 μM	Aggregation of **F4b** upon coordination with citrate leading to emission turn-on due to AIE	[123]
**F5**	418	~625	-	-	Yellowish-green, turn-on	0.35 μM	Coordination of citrate to Zn(II) centre weakens its fluorescence quenching effect on **F5**. Aggregation of **F5**-citrate complex led to emission enhancement.	[124]
**F6**	~305	485 (aggr) ^f^	-	-	Blue, enhanced	0.1 μM	Aggregation of **F6** upon coordination with citrate leading to emission turn-on due to AIE	[125]
*Anionic Surfactant Sensors*								
**F7**	-	324, 484	55 (aggr) ^f^	-	Blue, turn-on	-	Aggregation of **F7** leading o emission turn-on at surfactants’ CMC.	[126]
**F8**	286, 334	450 (aq.) ^e^; 510 (solid) ^g^	0.2 (aq) ^e^; 8.5 (+SDBS)	-	Green, turn-on	0.051 μM	Micelles formation leading to RIR in **F8** hence emission turn-on	[127]
**F9**	320 (soln) ^e^; 425 (aggr) ^f^	650 (soln) ^e^; 600 (aggr) ^f^; 650 (+SDS)	1.4 (soln) ^e^; 16 (aggr) ^f^	-	Red, turn-on	48 nM	Aggregate formation between AIE-active **F9** and SDS caused emission turn-on	[128]

^a^ Absorption maxima; ^b^ Emission maxima; ^c^ Fluorescence quantum yield; ^d^ Limit of Detection; ^e^ (soln/aq.) denotes optical properties in solution/aqueous state; ^f^ (aggr) denotes optical properties of aggregates; ^g^ (solid/film/gel) denotes optical properties in solid/film/gel state; ^h^ (+X^n+/−^) denotes optical properties in the presence of ion X^n+/−^. “-”: data were not reported.
